# Multi-omic approach to characterize the venom of the parasitic wasp *Cotesia congregata* (Hymenoptera: Braconidae)

**DOI:** 10.1186/s12864-025-11604-y

**Published:** 2025-04-30

**Authors:** Sébastien J. M. Moreau, Lorène Marchal, Hélène Boulain, Karine Musset, Valérie Labas, Daniel Tomas, Jérémy Gauthier, Jean-Michel Drezen

**Affiliations:** 1 Institut de Recherche sur la Biologie de l’Insecte, UMR 7261 CNRS, Université de Tours, Tours, 37000 France; 2 Department of Ecology and Evolution, University of Lausanne, Lausanne, 1015 Switzerland; 3 PRC, INRAE, CNRS, Université de Tours, Nouzilly, 37380 France; 4 Plateforme de Phénotypage par Imagerie in/eX Vivo de L’ANImal À La Molécule (PIXANIM), INRAE, Université de Tours, CHU de Tours, Nouzilly, 37380 France; 5 Naturéum - Cantonal Museum of Natural Sciences, Lausanne, 1005 Switzerland

**Keywords:** *Cotesia congregata*, Transcriptomics, Proteomics; Braconidae; Parasitoid, Venom

## Abstract

**Background:**

*Cotesia congregata* is a parasitoid Hymenoptera belonging to the Braconidae family and carrying CCBV (*Cotesia congregata* Bracovirus), an endosymbiotic polydnavirus. CCBV virus is considered as the main virulence factor of this species, which has raised questions, over the past thirty years, about the potential roles of venom in the parasitic interaction between *C. congregata* and its host, *Manduca sexta* (Lepidoptera: Sphingidae). To investigate *C. congregata* venom composition, we identified genes overexpressed in the venom glands (VGs) compared to ovaries, analyzed the protein composition of this fluid and performed a detailed analysis of conserved domains of these proteins.

**Results:**

Of the 14 140 known genes of the *C. congregata* genome, 659 genes were significantly over-expressed (with 10-fold or higher changes in expression) in the VGs of female *C. congregata*, compared with the ovaries. We identified 30 proteins whose presence was confirmed in venom extracts by proteomic analyses. Twenty-four of these were produced as precursor molecules containing a predicted signal peptide. Six of the proteins lacked a predicted signal peptide, suggesting that venom production in *C. congregata* also involves non-canonical secretion mechanisms. We have also analysed 18 additional proteins and peptides of interest whose presence in venom remains uncertain, but which could play a role in VG function.

**Conclusions:**

Our results show that the venom of *C. congregata* not only contains proteins (including several enzymes) homologous to well-known venomous compounds, but also original proteins that appear to be specific to this species. This exhaustive study sheds a new light on this venom composition, the molecular diversity of which was unexpected. These data pave the way for targeted functional analyses and to better understand the evolutionary mechanisms that have led to the formation of the venomous arsenals we observe today in parasitoid insects.

**Supplementary Information:**

The online version contains supplementary material available at 10.1186/s12864-025-11604-y.

## Background

Parasitoid insects are defined as organisms that develop at the expense of another host organism, whose death results from a direct or indirect consequence of their development [1]. Among parasitoid species, parasitoid wasps (Hymenoptera) manipulate their host’s physiology using a diversified range of virulence factors including embryonic, larval and maternal factors. Embryonic factors include the use of specialized cells named teratocytes, originating from the extraembryonic serosa, which abundantly secrete proteins into the haemolymph of the parasitized host [2, 3]. The main larval factor is saliva [4, 5]. Maternal factors include ovarian proteins [6–10], symbiotic polydnaviruses (PDVs) or virus-like particles (VLPs) produced by endogenous viruses present in the genome of these insects [11–15]. Female wasps within the genus *Leptopilina* also produce extracellular vesicles formerly named VLPs, but now also designated by different authors as Mixed Strategy Extracellular Vesicles (MSEV) [16] or venosomes [17]. It is indeed a matter of debate whether the filamentoviridae endogenous virus present in the *Leptopilina* genomes [18] is involved in vesicle production, as only one virally derived protein could be identified as being part of these structures by proteomic analyses [19]. All hymenopteran parasitoid females inject venom in the parasitized host at the time of oviposition [20, 21], making venom gland (VG) secretions a very important category of maternal factors. After venom injection, one or several eggs are deposited outside (for ectoparasitoids) or inside (for endoparasitoids) the body of the insect host [22], depending on the parasitic lifestyle. In some cases, ovarian fluids antagonize the powerful effects of venom toxins that otherwise would prematurely kill the host [23, 24].

The composition and functions of venoms from ectoparasitoid and endoparasitoid species have received a growing interest thanks to major improvements in high throughput analyses of DNA, RNA and proteins which allow combined analytical approaches [25, 26].

Since 2015, venoms of over 45 parasitoid species have been thoroughly investigated by these means, allowing the description of the cocktail of peptides and proteins that enter in their composition. However, many questions remain unanswered, such as what are the mechanisms at the basis of VG cell secretion and what are the evolutionary forces that drive the evolution of the virulence factors contained within venom. It appears that the role and composition of venoms can greatly vary even between closely related species of parasitoid Hymenoptera [20]. In this regard, the *Cotesia* genus (Hymenoptera: Braconidae) is an interesting taxon to study with a great variation in the importance of venom for parasitic success depending on the species and in the functional diversification among venomous secretions. For example, in *Cotesia melanoscela*, the venom ensures virus uncoating and uptake of viral particles by host cells [27] whereas in *Cotesia glomerata*, it directly protects eggs from encapsulation by the hemocytes of the host *Pieris rapae* [28]. In *Cotesia rubecula*, the Vn4.6 venom polypeptide is known to interfere with the activation of the host hemolymph prophenoloxidase [29]. The venom of *Cotesia chilonis* inhibits host humoral immunity and synergizes the immunosuppressive effects of the calyx fluid produced at the basis of the ovaries of the female wasp [30]. In *Cotesia vestalis* (formerly named *Cotesia plutellae*), the venom also synergizes the immunosuppressive effect of calyx fluid or PDVs and has a transient effect, at high doses, on the spreading and survival of *Plutella xylostella* plasmatocytes [31].

By contrast, Beckage et al. stated in 1994 that host envenomation was not required for the parasitic success of *Cotesia congregata*, since eggs experimentally injected with PDVs alone into caterpillars of its host, the tobacco hornworm *(Manduca sexta*), were able to develop successfully [32]. In addition, injection of venom alone in non-parasitized *M. sexta* larvae had no apparent effect on the levels of hemolymph proteins, larval growth and metamorphosis [32]. However, the production of a mix of active substances likely represents a significant physiological cost for *C. congregata* females. It is hence doubtful that this complex arsenal selected during evolution has no effect on parasitism success [33]. Subtle effects may have been overlooked due to methodological limitations or biases in the experimental design of previous physiological studies. To provide further clues on the potential role of *C. congregata* venom, we investigated its protein composition by combining genomic, transcriptomic and proteomic approaches. In the present paper a brief anatomical description of the venom apparatus is given, using fluorescent microscopy and confocal imaging, followed by a detailed list of the main venomous components. Our results are discussed in the light of knowledge gained on the composition of venoms of hymenopteran parasitoids in the last ten years. Together, these new data pave the way for functional studies and the understanding of the evolutionary mechanisms that led to the formation of the venomous arsenals we observe today in modern Hymenoptera.

## Methods

### Rearings and sample preparation

The *C. congregata* laboratory strain was reared on its natural host, the tobacco hornworm, *M. sexta* (Lepidoptera: Sphingidae) fed using artificial diet as described previously [32, 34, 35].

Isolation of ovaries and VGs of *C. congregata* females in order to perform mRNA and protein analyses were performed as previously described [36]. Briefly, ovaries and VGs were extracted from females at emergence. Female wasps were anesthetized on ice for several minutes, shortly rinsed in 70% ethanol and air dried. The abdominal organs, including ovaries and venom apparatuses, were gently pulled out with forceps and placed in 50 μl sterile Insect Ringer (for RNA extraction and microscopy) or sterile water (for collection of venom extracts).

### Fluorescence microscopy and confocal microscopy imaging of VGs

Observation of VGs under confocal microscopy was performed according to the protocol published by Cambier and collaborators [37].

### RNA extraction and RNA-seq analysis

Two replicates of 20 pairs of ovaries and 100 pairs of VGs were dissected and pooled together. RNA extractions were performed and RNA-Seq library preparations were carried out from 1 to 2 μg total RNA as described in [36]. Each library was sequenced using 100 bp paired-end reads on a HiSeq2000 Illumina sequencer. A total of 19.2 Gb were sequenced for the four libraries with an average of 48.2 million reads per library (sd = 6.2 millions). The paired-end reads from *C. congregata* ovary and VG libraries were mapped on the reference genome [36] using TopHat2 with default parameters [38] resulting in an average mapping percentage of 91.1% (sd = 5.6%) (detailed in [36]). The featureCounts program from the Subread package [39] was used to determine fragment counts per genes (default parameters) using the *C. congregata* OGS2.3_20170323 containing 14 140 genes and available at https://bipaa.genouest.org/sp/cotesia/.

To analyze gene expression the raw fragment counts of ovaries and VGs samples were first converted to counts per million (CPM) using the edge-R implemented package [40]. Statistical analysis was further performed following standard protocol as we previously described [36], including the edgeR TMM method for Normalization Factor calculation [41] and empirical Bayes quasi-likelihood F-tests to identify differentially expressed (DE) genes under chosen contrasts [42]. F-test *p*-values were adjusted using false-discovery rate (FDR) method [43]. When FDR was inferior to 0.001 and fold change (FC) of expressions between compared conditions was higher or equal to 2, genes were considered as DE. Genes with a significantly higher level of expression in VGs compared to ovaries were considered as putative venom genes.

### Collection of venom proteins

Thirteen newly emerged *C. congregata* females were anesthetized on ice and their venom apparatus (two VG filaments and a central reservoir) was dissected in 50 μL of sterile milliQ water at 4 °C, under a Stemi stereomicroscope (Carl Zeiss Microscopy GmbH, Jena, Germany). To avoid the leakage of venomous fluid from the reservoir, each venom apparatus was first separated from the venom duct (*ductus venatus*) downstream from the reservoir’s distal end and transferred in another drop of cold 50 μL sterile water, using a thin entomological pin. Surrounding fat tissues were carefully removed before the transfer of the venom apparatus in 15 μL of sterile milliQ water, kept on ice, with a sterile thin pin. The reservoir was then gently pressed with the pin to allow venom to diffuse in water, and the organ was immediately removed with the pin. When venom was extracted from the 13 venom apparatuses, the extract was centrifuged (1000 g, 5 min, 4 °C) and the supernatant was recovered and stored 15 h at -20 °C. Protein concentration of the venom extract was determined in triplicates by spectrophotometry according to Bradford method [44].

To visualize the protein profile by SDS-PAGE, 7 μL of venom extract containing 10 μg of total proteins were mixed with 2 μl of 5X concentrated Laemmli sample buffer (0.225 M Tris-HCl pH 6.8, 12% (w/v) SDS, 50% (v/v) glycerol, 0.5% bromophenol blue, 5% (v/v) b-mercaptoethanol) and 1 μL milliQ water and heated at 96 °C for 5 min. Fractionation of proteins was performed using a 12,5%Tris-SDS gel or a 15%Tris-SDS gel [see Additional file 1]. Electrophoresis was performed in 0.025 M Tris, 0.2 M glycine, and 0.1% (w/v) SDS. Staining of the gel was done overnight by soaking the gel in a staining solution (0.1% (w/v) Coomassie Brilliant Blue R-250 (MP Biomedical) in 50% (v/v) ethanol and 10% (v/v) acetic acid), followed by several baths of destaining solution (20% (v/v) ethanol and 7.5% (v/v) acetic acid) to destain the gel. Destaining was stopped with milliQ water.

For protein identification by GeLC-MS/MS (protein samples included in polyacrylamide gel and analyzed by nanoLC–MS/MS after in-gel digestion), venom extract was included in a 12,5%Tris-SDS polyacrylamide gel without fractionation electrophoresis using a constant voltage of 70 V in the stacking gel and 100 V in the running gel, for only 5 min. After staining with Coomassie Brilliant Blue R-250, the single band was excised and transferred into an Eppendorf tube and stored at -20 °C.

### Analysis of venom proteins by tandem mass spectrometry

The gel band was cut and in-gel digestion step was performed as previously described [45]. The resultant peptide mixture was analyzed by on-line nanoflow liquid chromatography tandem mass spectrometry (nanoLC-MS/MS) at the PIXANIM platform (INRAE, Nouzilly, France) as previously described [46]. Briefly, all experiments were performed on a LTQ Velos ETD Orbitrap Mass Spectrometer coupled to an Ultimate® 3000 RSLC Liquid Chromatographer (Thermo Fisher Scientific, Bremen, Germany) controlled by Chromeleon Software (v 6.80 SR13).

Samples were concentrated on a trap column (Acclaim PepMap 100 C18, 75 μm inner diameter x 2 cm long, 3 μm particles, 100 Å pores) and separated on a nano-column (Acclaim PepMap C18, 75 μm inner diameter x 50 cm long, 2 μm particles, 100 Å pores) at 300 nL/min. Mobile phases consisted of (A), 98% water, 2% acetonitrile in presence of 0.1% formic acid and (B) 20% water, 80% acetonitrile in presence of 0.1% formic acid. The gradient profile was as follows: (i) Equilibration of the columns with 96% solvent A and 4% solvent B; (ii) Gradient from 4 to 55% solvent B in 120 min; (iii) Step up to 99% solvent B for 15 min. Data were acquired in positive data-dependent mode using an Orbitrap resolution at 60,000. In the 300–1800 m/z range, the 20 most intense multicharged peptide ions were sequentially isolated (isolation width 2 m/z, 1 microscan) and fragmented in the trap using collision induced dissociation ion mode (collision energy at 35%, activation time 10 ms, Qz 0.25). Dynamic exclusion was activated (30 s with a repeat count of 1). A lock mass was enabled using the polydimethylcyclosiloxane ions (m/z 445.120025) for internal recalibration of the mass spectra.

MS/MS ion searches were performed using Mascot search engine version 2.7.0.1 (Matrix Science, London, UK) via Proteome Discoverer 2.5 software (ThermoFisher Scientific, Bremen, Germany) against a local database comprising all the predicted amino acid sequences deduced from the genome of *C. congregata*. The parameters used for database searches include trypsin protease with two missed cleavages allowed, carbamidomethylation, methionine oxidation and N-terminal protein acetylation as variable modifications. The error tolerance of the ions was set to 5 ppm for precursor and 0.8 Da for fragment ion matches. Results obtained from the target-decoy database searches were incorporated to Scaffold Q + S software (version 5.2.2, Proteome Software) [47] and were validated by the “Peptide Prophet” and “Protein Prophet” algorithms at the level of unique peptide with a protein identification probability at 99%.

### Sequence analysis

Nucleotide sequences of putative venom genes have been automatically annotated previously [36]. The automated annotations were followed by manual curations, corrections and expert annotations. Similar sequences were retrieved by comparing the sequences of interest with NCBI non redundant database with the BLASTP.

The signalP 6.0 algorithm [48] was accessed online [49] to predict the presence of five types of signal peptides, with the “Other” parameter selected. Functional annotations of the deduced amino acid sequences of all the putative venom genes were performed using the InterPro web site [50] that allows classification of submitted sequences in protein families and detailed sequence analyses by a set of specialized algorithms [51]. Sequences of proteins of unknown function were submitted to the Eukaryotic Linear Motif (ELM) server [52, 53] and to the Phyre2 web portal [54]. Other internet portals and databases were used for sequence analyses including Prosite [55, 56], Pfam [57, 58], PRINTS [59, 60], PANTHER [61, 62], GenBank [63, 64] and ParWaspDB [65].

Theoretical pI and Mw of each protein were calculated using the Compute pI/Mw online program [66–68]. Differences between groups of proteins with respect to their probabilities to possess a SP, their theoretical pI and Mw and the levels of overexpression of the corresponding genes were statistically tested using the Mann-Whitney U test, the Kruskal-Wallis one-way ANOVA test and the contingency Chi^2^ test using the Tanagra complement for Excel [69], with alpha acceptance levels of statistical significance between 0.05 and 0.1.

## Results

### Morphology of the venom apparatus of *C. congregata*

**Fig. 1:**
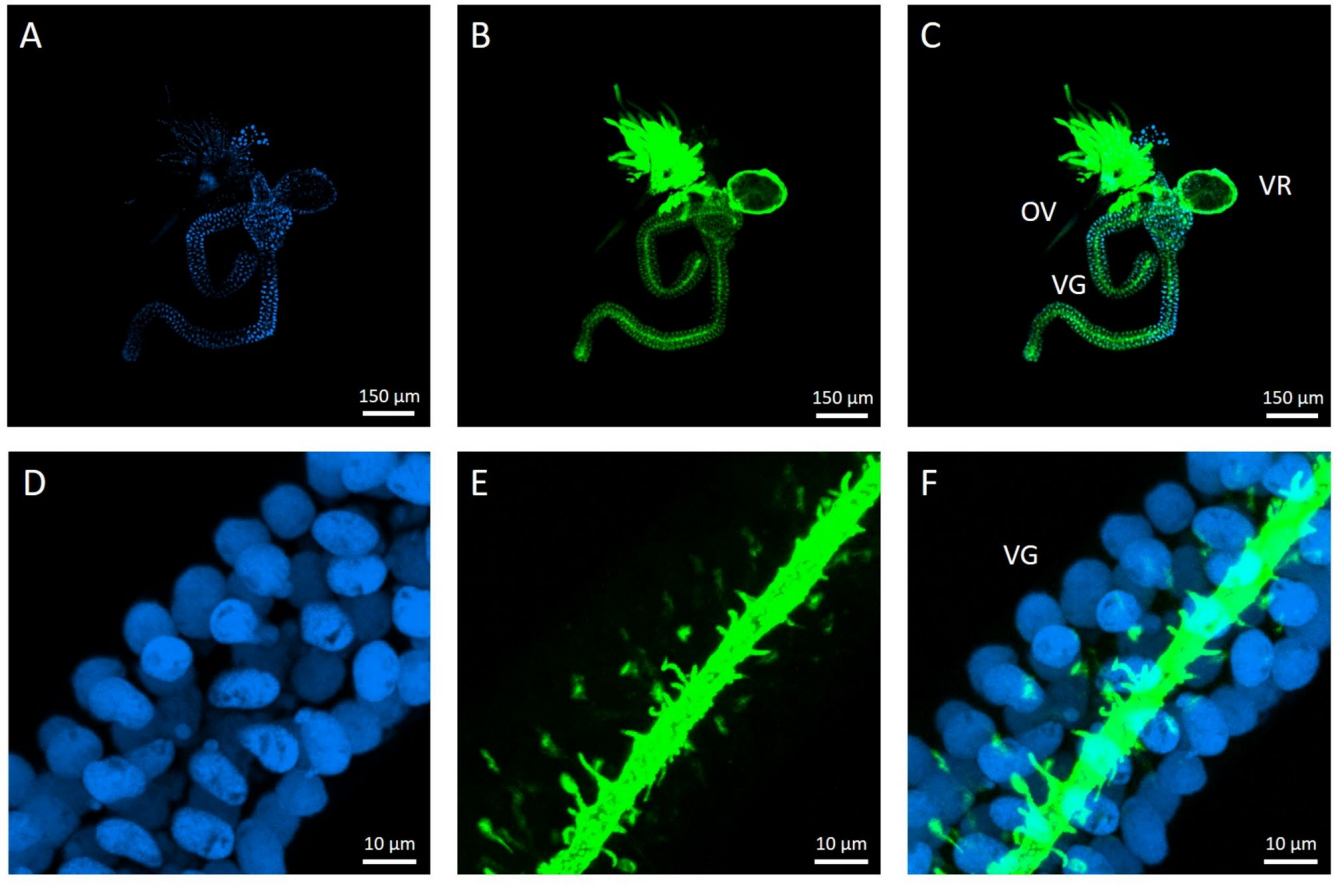
The venom apparatus of a female *C. congregata* observed in confocal microscopy imaging. **A**: Observation of nuclei using a 330–380 nm filter after DAPI staining; **B**: observation of chitin and actin-associated molecules using a 465–495 nm filter after FITC staining; **C**: merged picture of A and B. **D**-**F**: The venom gland at higher magnification. OV, Ovipositor; VG, venom gland; VR, venom reservoir. Pictures were taken using the 4x objective (A-C) or the 60x objective (D-F) of the confocal microscope

**Fig. 2:**
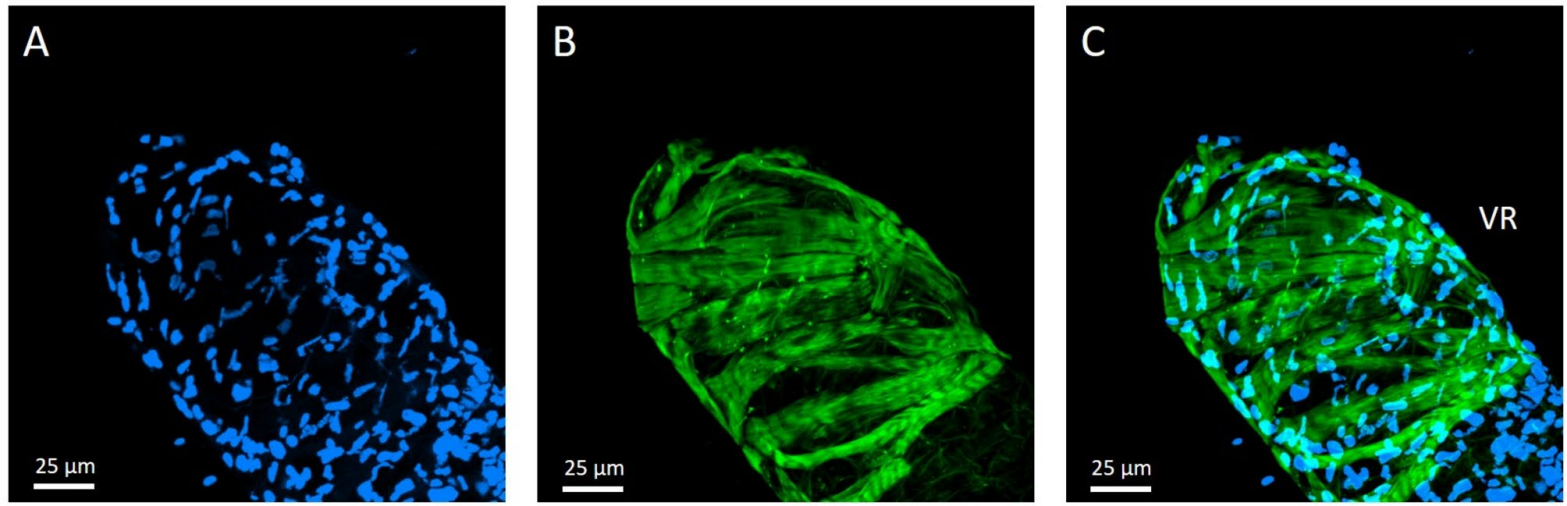
The venom reservoir of a female *C. congregata* observed in confocal microscopy imaging. **A**: Observation of nuclei using a 330–380 nm filter after DAPI staining; **B**: observation of chitin and actin-associated molecules using a 465–495 nm filter after FITC staining; **C**: merged picture of A and B. VR, venom reservoir. Pictures were taken using the 10x objective of the confocal microscope

The venom apparatus of *C. congregata* females is closely associated with the reproductive tract. It consists of a bilobed glandular system secreting venom in a central reservoir connected to the ovipositor *via* a short venom duct (Fig. 1A, B and C). Observed in confocal microscopy imaging using actin and DNA staining, the glandular cells appeared to be organized around a central chitin-lined collecting duct to which they are connected through small secretory ductules, also lined with chitin (Fig. 1D, E and F). The venom sac epithelia is surrounded by a loose network of striated muscular fibers, with no apparent glandular cells (Fig. 2A, B and C).

### Properties of venom proteins and relations with levels of genes expression

**Fig. 3:**
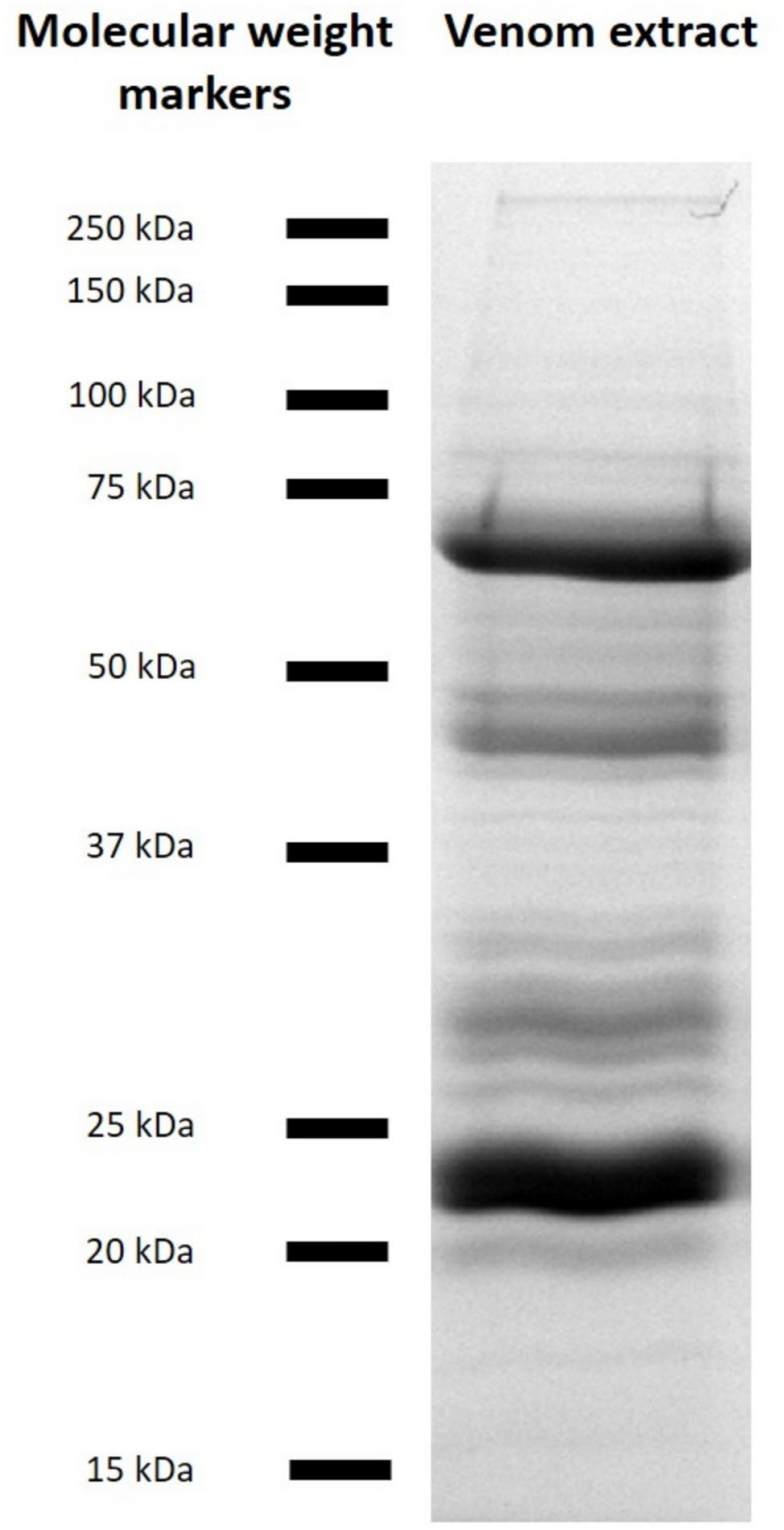
12.5% SDS-PAGE profile of *C. congregata* venom proteins (Coomassie Brilliant Blue staining). Positions of the molecular weight markers are indicated on the left

Thirty-one bands were observed on a 12.5% SDS-PAGE profile of a venom extract (Fig. 3) and on a 15% SDS-PAGE [see Additional file 1]. The apparent molecular weights of these denatured proteins ranked from 11 kDa to more than 250 kDa. Some bands could contain several proteins and some proteins could be composed of several subunits or isoforms leading to several bands. To go further and solve the venom composition of females *C. congregata*, we combined proteomic and transcriptomic analyses.

Overall, 659 genes were differentially expressed and upregulated (fold changes of expressions higher or equal to 10) in the VGs of females *C. congregata* compared to their ovaries, over a set of 14 140 genes. Conversely, 1881 genes were differentially expressed and upregulated in ovaries. A number of genes (8475) were similarly expressed in both organs, while 3125 genes were neither expressed in VGs nor in ovaries at detectable levels.

**Fig. 4:**
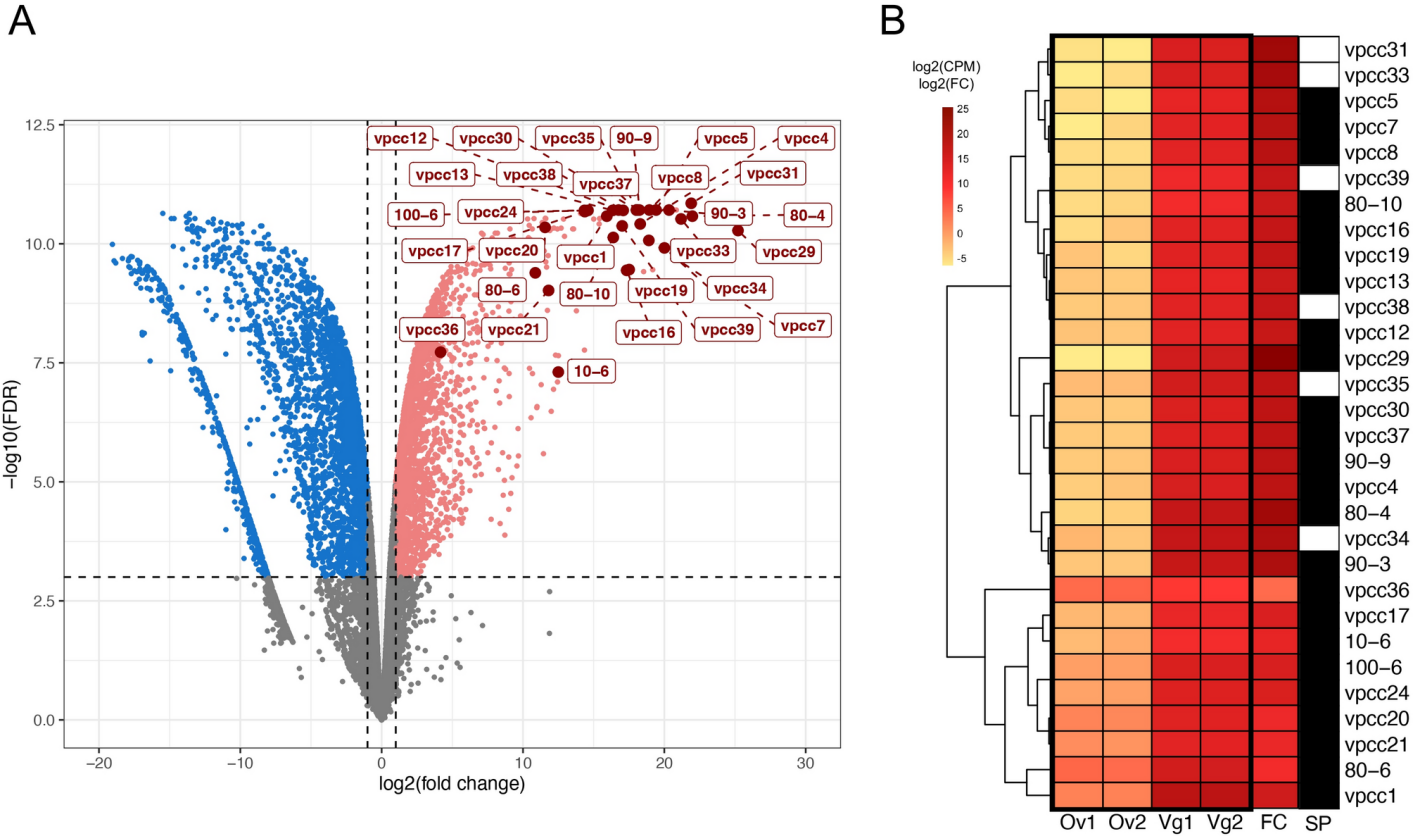
Expression patterns of 30 genes identified via proteomics of pure venom. **A**: Volcano plot of differentially expressed genes (DEGs) between ovaries (Ov) and venom glands (Vg). Genes up-regulated in ovaries and VGs are highlighted in blue and pink, respectively. The thresholds for significant differential expression are a fold-change (FC) > 2 (horizontal dashed lines) and FDR ≤ 0.001 (vertical dashed lines). Among the VG-upregulated genes, those encoding the 30 proteins identified in pure venom are specifically highlighted. **B**: Heatmap of expression levels for the 30 venom genes. Expression is shown as counts per million (CPM) across VG (Vg1, Vg2) and ovary (Ov1, Ov2) samples. Genes are hierarchically clustered based on their expression levels and FC between VGs and ovaries. Genes encoding venom proteins with predicted signal peptides (SP) are marked in black, while the six venom proteins lacking SP are shown in white

Among the 659 genes differentially expressed in VGs, we have analyzed a set of 30 genes encoding proteins and polypeptides (Fig. 4; Table 1) that were all detected in venom extracts by proteomic analysis [see Additional file 2]. Among them, we found 24 “venom proteins with SP” whose precursor forms all contained a predicted signal peptide (SP) and six “venom proteins devoid of SP” [see Additional file 3]. Concerning the second group of proteins, their corresponding genes were all overexpressed by VGs compared to ovaries: the levels of expression observed in VGs were 133 to 3.9 million-fold higher than in ovaries. However, none of them possessed a predicted SP, suggesting that these proteins used a divergent SP or a non-conventional transport pathway to be secreted in the venom of *C. congregata*. Three of them (vpcc35, vpcc38, vpcc39) were similar to proteins with predicted functions while the remaining three proteins (vpcc31, vpcc33, vpcc34) were of unknown function.

**Table 1:** Venom proteins of *C. congregata* and other proteins and peptides overexpressed by venom glands

Category of venom protein	Name	Genbank accession number	Sub-category	Sequence length (number of amino acids)	Theoretical molecular weight (Da)	Theoretical pI	Level of overexpression in venom glands compared to ovaries (Fold change)	Presence of a predicted signal peptide	SignalP 6.0 score for “Other”	SignalP 6.0 score for SP	Cleavage site position and probability	Type of SP
Venom proteins with predicted functions	10-6	CAD6231753.1	Histidine phosphatase superfamily	374	43748.95	5.61	5788.763269	yes	0.000267	0.999725	CS pos: 21–22. Pr: 0.9745	Sec/SPI
Venom proteins with predicted functions	80-6	CAD6211813.1	Serine carboxypeptidase (peptidase S10 family)	415	47499.92	5.58	1872.589864	yes	0.000281	0.999701	CS pos: 21–22. Pr: 0.9727	Sec/SPI
Venom proteins with predicted functions	80-10	CAD6208677.1	Pheromone/general odorant binding proteins	193	22119.92	4.52	85713.92416	yes	0.000197	0.999809	CS pos: 20–21. Pr: 0.9793	Sec/SPI
Venom proteins with predicted functions	90-3	CAD6208091.1	RAD52 family member	211	23930.92	5.16	1319547.77	yes	0.000262	0.999718	CS pos: 21–22. Pr: 0.9776	Sec/SPI
Venom proteins with predicted functions	100-6	CAD6225915.1	Neprilysin (peptidase M13 family)	701	82095.43	4.98	24765.48824	yes	0.000350	0.999615	CS pos: 26–27. Pr: 0.9127	Sec/SPI
Venom proteins with predicted functions	vpcc1	CAD6243299.1	5’-nucleotidase	584	65496.22	5.55	62362.20861	yes	0.000253	0.999743	CS pos: 21–22. Pr: 0.9723	Sec/SPI
Venom proteins with predicted functions	vpcc4	CAD6208700.1	Phosphodiesterases-like proteins of non eukaryotic origins	316	36482.74	6.37	321311.6661	yes	0.000000	1.000000	CS pos: 22–23. Pr: 0.9819	Sec/SPII (Lipoprotein signal peptide)
Venom proteins with predicted functions	vpcc5	CAD6239998.1	Pheromone/general odorant binding proteins	147	16650.67	4.52	699840.3707	yes	0.000200	0.999777	CS pos: 23–24. Pr: 0.5634	Sec/SPI
Venom proteins with predicted functions	vpcc7	CAD6242938.1	Proteins similar to bracoviral proteins	117	13200.18	5.00	490389.0837	yes	0.000288	0.999671	CS pos: 23–24. Pr: 0.5620	Sec/SPI
Venom proteins with predicted functions	vpcc8	CAD6242940.1	Proteins similar to bracoviral proteins	121	13091.98	7.71	503132.3393	yes	0.000640	0.999343	CS pos: 23–24. Pr: 0.7865	Sec/SPI
Venom proteins with predicted functions	vpcc12	CAD6204022.1	Phosphodiesterases-like proteins of non eukaryotic origins	329	38618.35	8.70	111204.8963	yes	0.000297	0.999687	CS pos: 25–26. Pr: 0.9754	Sec/SPI
Venom proteins with predicted functions	vpcc13	CAD6204024.1	Phosphodiesterases-like proteins of non eukaryotic origins	325	37596.77	8.54	83137.64966	yes	0.000410	0.999580	CS pos: 25–26. Pr: 0.9739	Sec/SPI
Venom proteins with predicted functions	vpcc17	CAD6208082.1	β-hexosaminidase	594	67858.23	8.10	21165.55925	yes	0.000260	0.999736	CS pos: 16–17. Pr: 0.9788	Sec/SPI
Venom proteins with predicted functions	vpcc19	CAD6231748.1	Histidine phosphatase superfamily	378	44381.65	5.97	186909.9505	yes	0.000340	0.999641	CS pos: 21–22. Pr: 0.9738	Sec/SPI
Venom proteins with predicted functions	vpcc24	CAD6204019.1	Phosphodiesterases-like proteins of non eukaryotic origins	327	37001.13	7.62	20833.39251	yes	0.000394	0.999600	CS pos: 25–26. Pr: 0.9686	Sec/SPI
Venom proteins with predicted functions	vpcc29	CAG5101080.1	Trypsin-like serine protease (peptidase S1 family)	248	28531.61	5.20	38859971.48	yes	0.000249	0.999716	CS pos: 18–19. Pr: 0.9781	Sec/SPI
Venom proteins with predicted functions	vpcc36	CAD6216008.1	Apolipoprotein D/lipocalin	193	21489.05	4.55	17.98939646	yes	0.000201	0.999783	CS pos: 19–20. Pr: 0.9799	Sec/SPI
Venom proteins of unknown functions	80-4	CAD6221875.1	Protein with no predicted functional domain	99	11183.81	6.86	4142901.67	yes	0.000184	0.999804	CS pos: 18–19. Pr: 0.9812	Sec/SPI
Venom proteins of unknown functions	90-9	CAD6222053.1	Protein with a predicted collagenase-like metallopeptidase catalytic domain	299	34573.66	8.10	308360.539	yes	0.002927	0.997030	CS pos: 20–21. Pr: 0.9105	Sec/SPI
Venom proteins of unknown functions	vpcc16	CAD6242748.1	Protein with no predicted functional domain	275	31717.19	4.71	165169.1627	yes	0.350858	0.649131	CS pos: 22–23. Pr: 0.4392	Sec/SPI
Venom proteins of unknown functions	vpcc20	CAD6245297.1	Proteins with tandem repeat DM9 domains	410	45920.01	6.38	3022.723265	yes	0.000244	0.999753	CS pos: 19–20. Pr: 0.9483	Sec/SPI
Venom proteins of unknown functions	vpcc21	CAD6245301.1	Proteins with tandem repeat DM9 domains	412	46122.41	7.32	3593.906156	yes	0.000245	0.999739	CS pos: 19–20. Pr: 0.9489	Sec/SPI
Venom proteins of unknown functions	vpcc30	CAD6240076.1	Proteins of the DUF4803 family	358	41279.76	8.28	266505.9691	yes	0.211360	0.788612	CS pos: 27–28. Pr: 0.7100	Sec/SPI
Venom proteins of unknown functions	vpcc37	CAD6224830.1	Cystein-rich protein	363	41392.66	6.62	268621.1561	yes	0.000183	0.999799	CS pos: 21–22. Pr: 0.9842	Sec/SPI
Venom proteins devoid of SP	vpcc31	CAD6240116.1	Proteins of the DUF4803 family	389	44230.11	8.16	3917693.501	no	1.000000	0.000001	NA	none
Venom proteins devoid of SP	vpcc33	CAD6240120.1	Proteins of the DUF4803 family	343	39144.95	5.57	2381275.61	no	0.999886	0.000133	NA	none
Venom proteins devoid of SP	vpcc34	CAD6211217.1	Protein with no predicted functional domain	141	16204.26	4.63	1053121.155	no	1.000000	0.000000	NA	none
Venom proteins devoid of SP	vpcc35	CAD6204869.1	Protein with serpin domains	400	45358.57	8.96	274982.7805	no	0.970902	0.029124	NA	none
Venom proteins devoid of SP	vpcc38	CAD6218065.1	Proteins with metalloprotease domains	474	54694.26	8.40	139367.6789	no	0.955623	0.044395	NA	none
Venom proteins devoid of SP	vpcc39	CAD6233576.1	Proteins with metalloprotease domains	546	64026.85	6.63	132722.9813	no	0.999977	0.000066	NA	none

Concerning the first set of 24 “venom proteins with SP”, 17 venom proteins with predicted functions were identified, including 10-6, 80-6, 80-10, 90-3, 100-6, vpcc1, vpcc4, vpcc5, vpcc7, vpcc8, vpcc12, vpcc13, vpcc17, vpcc19, vpcc24, vpcc29 and vpcc36. The genes encoding these proteins were all overexpressed by VGs, with observed levels of transcript production being 17.99 to almost 39 million-fold higher in VGs compared to ovaries (Table 1).

Within the group of the 24 “venom proteins with SP” also figured a set of 7 proteins of unknown or undefined functions including 80-4, 90-9, vpcc16, vpcc20, vpcc21, vpcc30 and vpcc37 [see Additional file 3]. They possessed predicted SPs and their presence in venom was confirmed by proteomic analysis [see Additional files 2 and 3]. Their respective genes were highly expressed by VGs, with levels of transcript production being 3023 to 4143-fold higher compared to those observed in the ovaries of *C. congregata* females.

In addition to the 30 venom proteins of *C. congregata*, 14 “putative venom proteins and peptides” were characterized [see Additional files 4 and 5]. With the exception of 80-5b, they all possessed a predicted SP, but their presence in venom was not confirmed by proteomic analyses. The genes coding for these proteins and peptides were all significantly overexpressed in VGs (from 14.09 to 1 693 151.32-fold higher than in ovaries). Their presence in venom, although plausible, has not been experimentally established. This group included eight proteins similar to proteins with predicted functions (vpcc2, vpcc10, vpcc11, vpcc18, vpcc22, vpcc23, vpcc28, vpcc40) and six peptides of unknown function (80-5a, 80-5b, vpcc6, vpcc9, vpcc14, vpcc15).

Finally, we analyzed four “products of genes overexpressed by VGs”, devoid of SP and not detected in venom [see Additional files 4 and 5]: vpcc27, vpcc41, vpcc25 and vpcc26. The levels of expression of these genes in VGs were 132 to 1633-fold higher than in ovaries. Upon sequence analysis and comparison, some functions could be assigned to vpcc27, vpcc41 and vpcc25 but not to the vpcc26 peptide.

The levels of overexpression greatly varied among genes encoding the 30 venom proteins of *C. congregata* (Fig. 4). A small subset of genes (6 out of 30 genes, hereafter designed as “massively expressed genes”) reached impressive levels of differential expression between VGs and ovaries exceeding 1 million-fold. Thirteen additional genes (“highly expressed genes”) had levels of expression comprised between 100 and 700 thousand-fold the expression observed in ovaries, while the remaining 11 genes (“overexpressed genes”) were overexpressed between 18-fold and 86 thousand-fold more in VGs than in ovaries. The distribution of the 30 genes between these three categories differed significantly according to the probability that the corresponding venom protein possessed or not a predicted SP (Chi^2^ test value = 6.2, *p-value* = 0.045). The proteins possessing a predicted SP were encoded by genes exhibiting levels of overexpression that were significantly higher (1 998 172.53 ± 7897 706.92-fold the level of expression observed in ovaries, in average) than those of genes encoding venom proteins devoid of any SP (1 316 527.26 ± 1 538 898.93) (Mann-Whitney U test, U = 39, *p-value* = 0.08709, α = 0.1). More information about the clustering of RNA samples from ovaries and VGs and expression levels of each gene corresponding to venom proteins, putative venom protein or peptides or gene products overexpressed by VGs is given in additional material [see Additional files 6 and 7].

Nineteen out of the 30 identified venom proteins had a theoretical isoelectric point (pI) below 7 and the average theoretical pI was 6.48 ± 1.48. The average theoretical molecular weight (Mw) of these venom proteins was 38 521.41 ± 17 322.50 Da, but we could not include in our proteomic analysis peptides below 11 000 Da for technical reasons. Indeed, venom proteins were submitted to a short SDS-PAGE run and analyzed from a gel slice cut out from this gel. Most peptides were likely lost during the run. Thus, the average Mw of the proteinic and peptidic fraction of *C. congregata* venom is probably inferior to this value. Acidic venom proteins had a slightly higher probability to possess a SP (0.82 ± 0.37) than basic venom proteins (0.71 ± 0.45) (Mann-Whitney U test, U = 56, *p-value* = 0.03686).

### Functional predictions inferred from 17 sequence analyses

We will introduce and discuss hereafter the possible functions of 17 proteins, whose presence in the venom of *C. congregata* females was confirmed by proteomic analysis, with respect to the most recent knowledge acquired on their respective families or class of molecules. When possible, we extended the functional annotation to similar proteins expressed by the VGs of other parasitoids, in order to compare their structures and to infer putative functions. Another group of 13 confirmed venom proteins of unknown function and/or devoid of SP was analyzed and discussed in detail in the supplementary materials [see Additional file 3]. An additional set of 18 putative venom proteins, peptides and interesting gene products, whose secretion could not be confirmed by proteomic analysis, was also analyzed in detail [see Additional file 4].

#### vpcc1

The vpcc1 (584 amino acids) venom protein belonged to the 5’-nucleotidase/apyrase enzyme family (InterPro: IPR006179). The sequence exhibited the conserved consensus pattern of 5’-nucleotidases ([LIVM]-x-[LIVM](2)-[HEA]-[TI]-x-D-x-H-[GSA]-x-[LIVMF]) between positions 29 and 41 of the mature protein. The vpcc1 protein exhibited 77.15 to 88.36% of sequence identity with 5’-nucleotidases predicted from the genomes of the parasitoids *Glyptapanteles indiensis* (GenBank: ABK56991.1), *Glyptapanteles flavicoxis* (GenBank: ACE75062.1) and *C. glomerata* (NCBI Reference Sequence: XP_044589411.1 and XP_044589412.1 and GenBank: KAH0547187.1), which correspond likely to homologous proteins of these closely related species.

#### vpcc19 and 10-6

Two proteins belonging to the histidine phosphatase superfamily were identified in the venom of *C. congregata*: the vpcc19 (378 residues) and the highly similar 10-6 (374 residues) protein. They shared 93% of sequence identity. They also shared 46% of sequence identity with vpcc18 (370 residues), a protein whose presence in venom has not been confirmed by our proteomic analysis. The catalytic domains of these three proteins contained five conserved residues (R10, H11, R14 and H264 of the mature proteins, respectively), forming a catalytic core conserved among enzymes of the second branch of histidine phosphatases superfamily.

#### 100-6

A neprilysin-2-like protein, the 100-6 protein (701 residues including a 26 amino acids SP), has been identified in the venom of *C. congregata*. Its sequence is the longest among the venom proteins of *C. congregata*. The 100-6 venom protein belongs to the Peptidase M13 family [70]. It possesses the two groups of highly conserved motifs forming zinc-binding domains, which characterize neprilysin (NEP) proteins [71, 72]: at positions 514 to 518 (HELSH corresponding to the HExxH conserved motif) and 575 to 579 (ENIAD, corresponding to the ExxxD conserved motif) of the mature protein. Interestingly, a third highly conserved consensus sequence of NEPs (NAY/FY) that mediates substrate or inhibitor binding, is modified in “NAMY” between positions 473 and 476 of the 100-6 mature venom protein. In contrast, a fourth conserved motif (CxxW), present at the C-terminal end of NEPs and critical to protein folding and maturation [73], is absent in the 100-6 protein.

#### vpcc29

The tertiary structure of the vpcc29 venom protein (248 amino acids) corresponded to a protein related to trypsin-like serine proteases, according to the Phyre2 web portal. It possessed a domain found in proteases belonging to the MEROPS peptidase family S1 (clan PA) (InterPro: IPR009003). However, the classic catalytic triad of known serine proteases (His57, Asp102 and Ser195 as in chymotrypsinogen A) [74] was modified in vpcc29 in a Leu-Val-Ser triad at positions 66, 112 and 210 of the precursor protein). Two important Gly residues out of three were conserved, however, in vpcc29 at positions 186 and 189. The only known sequences displaying a low but significant level of identity with vpcc29 (23.64 to 34.03% of sequence identity) were hypothetical or uncharacterized proteins from parasitoids of the Microgastrine subfamily including *C. glomerata* (GenBank: KAH0540702.1), *M. mediator* (GenBank: XP_057324437.1), *M. demolitor* (NCBI Reference Sequence: XP_008546250.1), *C. typhae* (GenBank: KAG8038842.1) and *C. chilonis* (GenBank: QBB01971.1). All these sequences were recognized as trypsin-like serine proteases by the Interproscan algorithm, except the one originating from *C. typhae* for which no protein family membership could be predicted.

#### 80-6

According to the InterPro prediction algorithm, the 80-6 venom protein (415 amino acids) belongs to the peptidase S10 family (InterPro: IPR001563), also known as carboxypeptidases C family. The alignment of the amino acid sequence of 80-6 with similar serine carboxypeptidases and comparison with the consensus patterns for S10 peptidases from the PROSITE and the PFAM databases (PROSITE: PDOC00122; PFAM: PF00450) allowed us to locate and analyze the residues forming the expected triad.

First, a serine to asparagine substitution at position 140 of the mature 80-6 protein distinguished 80-6 from the other S10 peptidases, in which the consensus sequence for the serine active site was [LIVM]-x-[GSTA]-E-*S*-Y-[AG]-[GS] (where *S* was the active site serine residue) (PROSITE: PS00131). A serine residue was still present in the modified domain of 80 − 6, but at position 138 of the mature protein (MM*S*ENVGT).

Second, the histidine active site (YYIIEAG*H*LLIVDNP between positions 367 and 381 of the mature 80-6 protein, where *H* would be the active site histidine residue) was also modified in comparison to the consensus sequence for the histidine active site of serine carboxypeptidases ([LIVF]-x(2)-[LIVSTA]-x-[IVPST]-x-[GSDNQL]-[SAGV]-[SG]-*H*-x-[IVAQ]-P-x(3)-[PSA]; PROSITE: PS00560). The first three amino acids of the consensus sequence were lacking. Four out of the fifteen remaining amino acids differed from the expected residues at the corresponding positions.

Finally, an aspartic acid residue possibly corresponding to an active site residue has been located at position 322 of the mature 80-6 protein. This residue aligned with an aspartic acid residue conserved in 82% of the sequences of serine carboxypeptidases which were used for the design of the PFAM signature of the peptidase S10 family (PFAM: PF00450; residue 344 of the sequence logo).

It is of note that none of these active sites were detected by the InterPro algorithm nor PROSITE Expasy algorithm: the algorithms correctly assigned the 80-6 protein to the peptidase S10 family but were unable to locate the residues of the conserved triad. This was probably due to the existence of subtle variations from the consensus patterns observed in 80-6. This protein shared 88.43% of sequence identity with Cc-Ven5 (GenBank: APD15616.1), a retinoid-inducible serine carboxypeptidase-like protein overexpressed by the VGs of *C. chilonis* [75].

#### vpcc5 and 80-10

The vpcc5 venom protein (147 amino acids) has no similar sequence in databases. However, according to the InterPro prediction program, its secondary structure corresponded to a protein belonging to the Pheromone/general odorant binding protein (P/GOBP) superfamily (InterPro: IPR036728).

In addition to vpcc5, the venom of *C. congregata* contained a second PBP/GOBP-like protein, the 80-10 protein (193 amino acids). This protein possessed 6 cysteine residues and only shared 11.9% of sequence identity with vpcc5. The 80-10 protein shared 61.66 to 68.64% of sequence identity with hypothetical proteins from *Cotesia flavipes* (GenBank: UEP64252.1), *C. typhae* (GenBank: KAG8040250.1) and *C. glomerata* (GenBank: KAH0555401.1).

#### vpcc7 and vpcc8

Vpcc7 (117 amino acids) and vpcc8 (121 amino acids) were two related venom proteins similar to a hypothetical protein of unknown function, CcBV_5.3 (BV16 Family 2 members), encoded by the genome of the *C. congregata* Bracovirus (CCBV) (NCBI Reference Sequence: YP_184787.1). They respectively shared 45.67% and 41.1% of sequence identity with CcBV_5.3 and 54% of sequence identity between them.

#### vpcc4, vpcc12, vpcc13 and vpcc24

The venom of *C. congregata* contained four members of the phospholipase C (PLC)-like phosphodiesterases superfamily (InterPro: IPR017946): vpcc4 (316 amino acids), vpcc12 (329 amino acids), vpcc13 (325 amino acids) and vpcc24 (327 amino acids). A gene encoding a fifth similar sequence, vpcc25 (346 amino acids), was overexpressed in the VGs compared to ovaries, but the presence of the corresponding protein in venom was not confirmed by proteomic analysis. The vpcc25 protein had a low probability to possess a signal peptide (0.594 out of a maximum score of 1), according to SignalP 6.0. The amino acid sequence of vpcc4 exhibited a lipoprotein signal peptide instead of a classical SP. Vpcc24 shared 79% of sequence identity with vpcc25, 68.6 and 62.5% with vpcc12 and vpcc13 and only 28.9% of sequence identity with vpcc4.

Interestingly, the vpcc4 venom protein was the only one to possess a phosphatidylinositol-specific phospholipase C (PI-PLC) X domain, extending from positions 42 to 159 of the mature protein. However, the Y domain, which constitutes the second characteristic functional domain of eukaryotic PI-PLCs, was lacking in the vpcc4 sequence, as it was in vpcc12, vpcc13 and vpcc24. The vpcc24 sequence exhibited 29.82 to 67.79% of sequence identity with sequences from Microgastrinae and 28.12 to 30.63% of sequence identity with bacterial PLC-like proteins.

#### vpcc17

The vpcc17 venom protein (594 amino acids) corresponded to a β-hexosaminidase (InterPro: IPR025705). The mature vpcc17 protein possessed the N-terminal domain of the eukaryotic β-hexosaminidases (PFAM domain: PF14845), from positions 44 to 164, and a TIM barrel (triose-phosphate isomerase) fold corresponding to a catalytic domain (PFAM domain: PF00728), between positions 188 and 536. Homologous proteins predicted from numerous genomes of Hymenoptera were returned by the BLASTP search using vpcc17 as the entry sequence. Among the matching gene products figured the sequence of Cc-Ven12 (GenBank: APD15623.1), a protein produced by the VGs of *C. chilonis* [75] that shared 86.2% of sequence identity with vpcc17. Vpcc17 also shared 14.5% of sequence identity with a β-hexosaminidase-like truncated protein (207 amino acids only, encoded by the sequence Dr_contig00438) that we have previously identified as a protein produced by the VGs of the cynipid gall wasp *D. rosae* [37].

#### 90-3

The 90-3 venom protein (211 amino acids) contained a dsRNA-binding domain extending from positions 41 to 162 and was recognized as a RAD52 family member by the InterPro algorithm (InterPro: IPR041247). The 90-3 protein shared 30.15 to 33.33% of sequence identity with proteins predicted as DNA repair and recombination proteins from *C. congregata* (GenBank: CAG5075450.1) and *C. glomerata* (NCBI Reference Sequence: XP_044588796.1, XP_044586548.1, XP_044596685.1, and GenBank: KAH0562764.1) that all lacked a predicted SP.

#### vpcc36

The vpcc36 venom protein (193 amino acids) exhibited 4 sequence signatures specific from apolipoprotein D-like proteins (PRINTS: PR01219), also named lipocalins (InterPro: IPR022271) at positions 19–33, 104–115, 148–164 and 174–193 of the precursor protein. The sequence contained a SP of 19 amino acids and 31 residues predicted to be involved in a ligand binding cavity of apolipoprotein D and similar proteins (Conserved Domains entry: cd19437).

## Discussion

### Protein richness of *C. congregata* venom

The venom of *C. congregata* contains at least 30 venom proteins. This number of venom proteins is close to those observed in other Braconid species: for example, VGs of *B. hebetor* females produce 27 main proteins [76] and females *Chelonus inanitus* produce 29 venom proteins [26]. Females *P. lounsburyi* and *P. concolor* produce 39 and 40 venom proteins, respectively [77]. This number is also globally in accordance with the 31 denatured proteins observed on the 12.5% SDS-PAGE profile (Fig. 3). It is likely that other *C. congregata* venom proteins remain to be identified, and it would be of particular interest to specifically study the venomous peptides of this parasitoid wasp with appropriate methods.

In most animals, the protein/mRNA ratio is constant across cell types and tissues but varies by several orders of magnitude from one gene to another. Therefore, protein abundance is not directly inferable from gene expression levels [78]. In parasitoid VGs however, the most expressed genes produce the most abundant venom proteins [20] and VGs generally produce a small number of highly abundant proteins and peptides and a large number of low abundance products. Our results confirmed that globally, the venom proteins corresponded to the most expressed genes in the VGs of *C. congregata*, which represent a small set of the 659 genes overexpressed by this tissue. Among these genes, we have identified four genes whose products (vpcc27, vpcc41, vpcc25 and vpcc26) are not part of the venom but could play a role in VG function and/or in the maintenance of its structure and integrity. They potentially encode crucial functions for the safe production and secretion of the venomous arsenal and to avoid nonintentional damages to the wasp’s tissues and organs. As only two replicates were performed for each tissue, the observed gene expression levels should not be taken as absolute and definitive values. They are likely to vary from one individual to another, and even at different stages in the development of parasitoid wasps. The average expression levels allowed to identify genes of interest that are over-expressed in VGs and to attempt to establish interesting relationships between these expression levels and certain characteristics of the deduced proteins.

In the past, some authors referred to the venom-secreting glands as “acid glands”, in contrast to Dufour’s “alkaline” gland, which produces marking pheromones [79–83]. Our results confirm that the majority of *C. congretata* venom proteins are acidic proteins and possess a SP allowing them to be secreted by a classical pathway. However, 3 out of the 6 genes with the higher levels of overexpression in VGs compared to ovaries, encoded venom proteins whose sequences were devoid of SPs. One of these venom proteins was a basic protein (vpcc31). This suggests that *C. congregata* use canonical and non-canonical secretory pathways to secrete venom proteins and notably a basic one. Therefore, our study demonstrates that not all venom proteins are acidic in nature, and not all have SP.

### Diversity and specificity of *C. congregata* venom hydrolases

Our study revealed that *C. congregata* venom is above all a diversified mixture of hydrolytic enzymes likely to interact with a wide range of molecules in the host and/or within the VG itself (see below). This may seem surprising in view of the previously published article on the supposed lack of involvement of this venom in the reproductive success of *C. congregata* [32]. However, our results are consistent with those from works carried out in recent years on other parasitoid species, and notably within the *Cotesia* genus. They show that the venom of these species is indeed a mixture of active substances, capable of directly provoking physiological effects in envenomated hosts or of facilitating the action of other virulence factors, like polydnaviruses. Sequence analysis and comparisons reveal subtle differences between *C. congregata* venom hydrolases and the hydrolytic enzymes of other parasitoids, which probably have functional consequences.

The 5’-nucleotidase/apyrase enzyme family, to which vpcc1 belongs, gathers ubiquitous proteins that hydrolyze phosphate esterified at carbon 5’ of 5-carbon sugars (ribose or deoxyribose) of nucleotide molecules. Hence, they are crucial for the degradation of nucleotides. In the braconid wasp *Meteorus pulchricornis* [84], a protein sharing low sequence similarity with bacterial and eukaryotic ecto-5’-nucleotidases was also strongly expressed by VGs. However, it lacked the above-mentioned 5’-nucleotidase signature found in vpcc1. A 5’-nucleotidase gene was also found overexpressed by the VGs of the cynipid gall wasp *Diplolepis rosae* [37].

The vpcc19 and 10-6 venom proteins belong to the second branch of histidine phosphatases superfamily. This group of sequences is notably composed of acid phosphatases that hydrolyse phosphate esters, optimally at low pH [85]. Acid phosphatases were identified in the venoms of several Hymenoptera, for instance in the Apidae *Apis mellifera* [86] and *Apis cerana* [87], the Ptromalidae *Nasonia vitripennis* [25] and *Pteromalus puparum* [88], the Ampulicidae *Ampulex compressa* [89], the Figitidae *Leptopilina boulardi* and *Leptopilina heterotoma* [90] and the Ichneumonidae *Pimpla hypochondriaca* [91]. Venom acid phosphatases generally act as spreading factors for other venom components.

The structural features of the 100-6 venom protein suggest a potential difference in the functioning of this venom enzyme compared to other known NEPs. Most NEPs are type II integral membrane proteins acting as oligopeptidases, but some family members correspond to soluble secreted proteins, such as in *Drosophila* [71, 72, 92]. Of note, a NEP is one of the major components of *Venturia canescens* VLPs [93] produced by an endogenous nudivirus [14] and protecting parasitoid eggs from encapsulation by host hemocytes. NEP-like proteins appear to be widely distributed among the venoms of Hymenoptera, except in the *Cotesia* genus. According to Colinet and collaborators [94], NEP-like proteins were indeed found in the venoms of Figitidae (*L. boulardi*), Braconidae (*M. demolitor*) and Ichneumonidae (*Hyposoter didymator*). NEP-like proteins were also reported from the venoms of *A. compressa* [89], *L. heterotoma* and a *Ganaspis* species (Figitidae) [90, 95], *T. nigriceps* [96], *Lysiphlebus fabarum* [97], *P. lounsburyi* and *P. concolor* (Braconidae) [77], *Tetrastichus brontispae* (Eulophidae) [98], *P. vindemmiae* (Pteromalidae) [99]. Yang and collaborators [100] have recently suggested that the recruitment events of venom NEP-like genes occurred independently during the radiation of Hymenoptera. In the dryinid wasp *Gonatopus flavifemur*, 7 NEP-like proteins, possessing predicted SPs, were found to be overexpressed at the mRNA level in VGs [100], but the presence of the corresponding proteins in the venom has not been verified by proteomic methods. A similar lack of information concerns two NEP-like genes expressed by the VGs of the Encyrtidae *Ooencyrtus telenomicida* [101] and the Braconidae *Microctonus hyperodae* [102]. Other NEP-like genes were expressed at low level in the VGs of the braconid wasps *Aphidius ervi* [94] and *Meteorus pulchricorni* [84]. The roles played by NEP-like proteins in host-parasitoid relationships remain to be clarified since experimental evidence is scarce. NEP are oligopeptidases with a wide range of biological activities and inferring some hypothetical roles from information available on mammal or *Drosophila* enzymes is quite difficult. However, it was shown experimentally that injections of a recombinant protein based on the sequence of Cp-NEP1, a NEP-like protein expressed in VGs of *C. vestalis*, disrupted the formation of melanized nodules against *Eschericha coli* in the host *Plutella xylostella* [103]. However, the fact that Cp-NEP1 lacks the two groups of highly conserved motifs (called “protein fingerprints”) found in most known NEPs casts doubt on the capacity of this protein to act as a classical soluble NEP, and therefore to generalize the observed effect to venom NEP-like proteins of other parasitoids, within or beyond the genus *Cotesia*. Interestingly, Teng and collaborators [75], who studied the venom of *C. chilonis*, did not mention the presence of a NEP-like protein in this fluid. This suggests that the presence of NEP-like proteins in the venom would not be a feature conserved in all *Cotesia* species.

The peptidase S1 family is known as the largest peptidase family, by both the number of sequenced proteins and the number of distinct peptidase activities. As a putative trypsin-like serine protease, vpcc29 belongs to this family. Three genes encoding trypsin-like proteins possessing predicted SPs were up-regulated in VGs of *C. vestalis*, compared to the remaining bodies of these females [104]. The modification of the classic catalytic triad of known serine proteases in vpcc29 suggest an ability to interact with specific peptidic ligands, either in the host or in the VG.

The 80-6 venom protein from *C. congregata* and Cc-Ven5, from *C. chilonis*, shared the same sequence features, and are thus probably homologous sequences: They belong to secreted serine carboxypeptidases, characterized by a catalytic triad including an aspartic acid residue, a histidine residue and a serine residue bonded together by two hydrogen bonds [105]. The sequences surrounding the serine and histidine active residues are highly conserved in all serine carboxypeptidases, while those near the aspartic acid active site are variable [105]. Consequently, consensus patterns are only available for two out of the three active sites of serine carboxypeptidases. Other serine carboxypeptidases of the S10 or S28 families (i.e. Lysosomal Pro-X Carboxypeptidase family) are expressed by the VGs of several Hymenoptera families including Braconidae [77, 96, 106], Apidae [107, 108], Cynipidae [37], Pteromalidae [109] and Formicidae [110, 111].

The group of venom proteins including vpcc4, vpcc12, vpcc13 and vpcc24 is related to the PLC superfamily but has diverged to such an extent that their sequence features distinguish them from classical eukaryotic phosphodiesterases. Intriguingly, the results of the BLASTP algorithm comparison of these sequences to the NCBI nr database revealed that the only sequences showing significant similarities with the four proteins originated either from other Microgastrinae parasitoids (including *C. glomerata*, *C. chilonis*, *C. typhae* and *Microplitis demolitor*) or from bacteria (including organisms of the class Bacilli, Flavobacteriia and Gammaproteobacteria). On the one hand, a first explanation for these results could be that these genes derive from a non-eukaryotic ancestor gene. Horizontal or lateral gene transfer from bacteria is an important route for metabolic innovation in insects [112]. In the gall wasp *Biorhiza pallida*, two cellulase genes expressed by the VG could have been acquired by horizontal transfer from bacteria [37]. Also, a GH19 chitinase gene originating from the unicellular microsporidia/Rozella clade was laterally transferred to parasitoid wasps of the Chalcidoidea lineage and the corresponding enzyme was recruited as a venom protein in at least 15 species of this family [113]. On the other hand, copies of cellular genes recruited as virulence factors by parasitoid females are often strongly divergent from the original sequences [114]. Bézier and collaborators [115] have even shown, in the case of the *C.congregata* polydnavirus CcBV, that a greater divergence level was a specific hallmark of the genes involved in the parasitoid virulence. Hence, it cannot be completely ruled out that sequence similarities found with bacterial sequences are due to both the extreme diversity and abundance of bacterial sequences in gene databases and to strong sequence divergence resulting from specific evolutionary constraints applying to these venom enzymes in the context of host-parasitic relationships. PLC activities are involved in some parasitoid-host relationships: for instance, *Galleria mellonella* larvae displayed an increase in PLC activity in hemocytes or fat body in response to the venoms of *Habrobracon hebetor* (synonym of *Bracon hebetor*) and *Habrobracon brevicornis* [116, 117]. This enhanced activity resulted in the death of the targeted cells. Furthermore, the venom of *N. vitripennis* induced a PLC transduction pathway dependent cell death in BTI-TN-5B1-4 cells, which could be transiently impaired by the use of PLC inhibitors [118].

The gene coding for vpcc17 belongs to a well conserved gene family even among distantly related hymenopteran species. Genes of this family code enzymes that hydrolyze the terminal non-reducing N-acetyl-D-hexosamine residues in N-acetyl-β-D-hexosaminides [119]. In the context of insect development, some β-hexosaminidases act as chitooligosaccharidolytic enzymes that are activated during metamorphosis to degrade chitin, an important component of insect exoskeletons. The host’s chitin is thus a plausible target of the vpcc17 venom protein.

### Potential roles of venom proteins with binding abilities

In addition to the previous hydrolases, *C. congregata* venom contains a set of proteins with binding abilities.

Like the vpcc5 and 80-10 proteins, several P/GOBPs, were already reported from venoms of parasitoid species: *N. vitripennis* [25], *C. inanitus* [26], *L. heterotoma* [120], *P. puparum* [121], *Anisopteromalus calandrae* [109], *Bracon nigricans* [76], *Torymus sinensis* [122] and *M. pulchricornis* [84]. Various roles have been suggested for these venom proteins, ranging from host selection to solubilization and transport of hydrophobic molecules [76, 122]. Vpcc5 only contained 4 cystein residues and therefore would belong to the Minus-C OBP family, whose members possess less than six cysteine residues [123, 124]. This feature is shared by the *B. nigricans* OBP-like venom protein [76].

In eukaryotes, the RAD52 proteins bind ssDNA and promote strand exchange via the pairing of complementary single strands [125, 126]. It seems that the unusual presence of a SP in frame with the sequence of a RAD52-like protein allowed the secretion of 90-3 by VGs of *C. congregata*. The ability of venom protein 90-3 to act on damaged DNA once injected into the host is difficult to hypothesize without the contribution of other proteins. If this were the case, it could contribute to the genome stability of different cell types (host tissues, embryonic cells and the extra-embryonic serosa of *C. congregata*, for example), to the benefit of the parasitoid.

The venom protein vpcc36 corresponds to an apolipoprotein D-like protein. Members of this family are characterized by several common molecular-recognition properties such as the ability to bind to small hydrophobic molecules and specific cell-surface receptors [127]. They can also form complexes with soluble macromolecules. They exhibit great functional diversity, ranging from pheromone transport to modulation of immune response [127]. Recently, an apolipoprotein D-like protein has been reported from the venom of the ant *Lasius flavus* (Hymenoptera: Formicidae) [128], but its role is unknown. We have previously reported a high production of apolipoproteins D by the VG of the gall wasp *B. pallida* [37] that very likely contributed to the high viscosity of the venom.

The venom of *C. congregata* also contained proteins of unknown function and proteins devoid of SP but exhibiting metalloprotease or serpin functional domains [see Additional file 3]. The VGs of *C. congregata* females overexpressed genes encoding several enzymes (a protein disulphide isomerase-like protein and a superoxide dismutase), proteins (calreticulin, proteins with cystatin-like domains or IAP-binding motif) and peptides whose presences in venom, although plausible, were not experimentally established [see Additional file 4]. Remarkably, the venom of *C. congregata* was apparently devoid of certain venomous compounds which are common in Hymenoptera, such as phospholipase A2, cathepsin-L or alkaline phosphatase, and that are over-expressed by the VGs of other *Cotesia* species like *C. vestalis* [104]. This is indicative of the numerous specialization processes that have taken place within the genus *Cotesia* during the course of evolution.

## Conclusions

In this paper, we report for the first time the identification of 30 venom proteins produced by the VGs of *C. congregata*, a braconid wasp that parasitizes caterpillars of *Manduca sexta* in laboratory and in field conditions. We have also identified several genes coding for putative venom proteins and peptides, although this latter category of small compounds was not initially targeted by our experimental design. Finally, we have characterized several gene products, overexpressed by VGs, that could correspond to proteins involved in VG function. Thank to meticulous sequence analyses and in silico functional predictions based on up-to-date algorithms, we were also able to describe the main sequence features of *C. congregata* venom proteins. This study paves the way to both future work in evolutionary biology and functional studies using parasitoid wasps as models.

We have observed the convergent recruitment of several protein groups already described in multiple venomous animal lineages [129] for use as venom components, like a neprilysin-like protein (the 100-6 protein), two acid phosphatases (vpcc19 and 10-6) and two P/GOBPs (vpcc5 and 80-10). We have also detected conserved venom proteins at the intrageneric (80-4) or intrafamily (vpcc29) levels, but also original proteins of unknown function that seem specific to *C. congregata* (vpcc7 and vpcc8). Interestingly, these latter two venom components showed sequence similarity with gene products encoded by the genome of the symbiotic bracovirus of *C. congregata.* In addition, four venom PI-PLCs from *C. congregata* (vpcc4, vpcc12, vpcc13 and vpcc24) lacked characteristic functional domains of eukaryotic enzymes and shared sequence similarity with gene products originating from bacteria. This raises the possibility that duplication of symbiotic polydnaviral genes, horizontal transfers and strong sequence divergence due to specific evolutionary constraints may have contributed to the current diversity of venom components in *C. congregata*.

The massive expression levels observed for some genes encoding venomous proteins confirmed that venom production represents a costly investment for *C. congregata* females. This finding contrasts with previous statements, based on convincing but limited physiological studies that suggested that the venom of *C. congregata* was not involved in the parasitic success of *C. congregata* eggs, once injected in their hosts. It is very unlikely that this mixture of hydrolytic enzymes and binding proteins plays no role in the parasitic interaction. The diversity and the effects of the venom proteins of these PDV-carrying wasps are probably linked to the diversity and the effects of polydnaviral gene products. Our work hence opens interesting perspectives for research, both to study the biological functions of *C. congregata* venom, and to understand the underlying evolutionary mechanisms that enabled the progressive elaboration of such a diversified arsenal.

## Electronic supplementary material

Below is the link to the electronic supplementary material.

Supplementary Material 1: Additional file 1: Original and unprocessed version of a 15% SDS-PAGE loaded with venom extracts of *C. congregata* at two different concentrations. Molecular weight markers are visible on the first and fourth line of the gel. Two samples of venom extracts, containing 10–14μg of proteins, are visible on the second and third lines of the gel

Supplementary Material 2: Additional file 2: Results of proteomic analysis. For each protein identified in the venom of *C. congregata*, the following information is given: total spectrum count, normalized spectral abundance factor (NSAF), total unique peptide count, total unique spectrum count and protein identification probability

Supplementary Material 3: Additional file 3: Description and analysis of venom proteins of unknown function and venom proteins devoid of SP. A set of 13 protein sequences deduced from genes overexpressed by VGs compared to ovaries is introduced and discussed. Secretion of these proteins by VGs was confirmed by proteomic analysis. Seven of these venom proteins possessed a predicted SP but their functions were unknown. Six of these proteins did not exhibit a predicted SP while a functional domain was found for three of them

Supplementary Material 4: Additional file 4: Description and analysis of putative venom proteins and peptides and products of genes overexpressed by venom glands. A selection of 18 additional protein sequences deduced from genes overexpressed by VGs compared to ovaries is introduced and discussed. Secretion of these proteins by VGs could not be confirmed by proteomic analysis. All putative venom proteins and peptides possessed a predicted SP, except 80-5b

Supplementary Material 5: Additional file 5: List of putative venom proteins and peptides and products of genes overexpressed by venom glands. Name, sequence length, theoretical molecular weight and isoelectric point, level of overexpression and SP prediction are given for each gene product

Supplementary Material 6: Additional file 6: Clustering of RNA samples from ovaries and venom glands. A: Multidimensional Scaling (MDS) plot showing the relationships between ovary (Ov) and venom gland (Vg) samples based on normalized expression data; B: Correlation heatmap illustrating pairwise sample correlations based on normalized expression data. The intensity of blue shading represents the degree of correlation, with darker shades indicating higher correlation

Supplementary Material 7: Additional file 7: Expression levels of 48 genes of interest in ovary (Ov1, Ov2) and venom gland (Vg1, Vg2) transcriptomes. raw_count: number of reads obtained from each sample; norm_cpm: counts per million; norm_cpm_av: average between the norm_cpm values of two replicates for each tissue (venom gland and ovary); Ov: ovary; Vg: Venom gland

## Data Availability

The accession numbers for raw data of RNAseq at wasp emergence are listed in the Bioproject PRJNA594477: SRX18987781 (https://www.ncbi.nlm.nih.gov/sra/SRX18987781[accn]) and SRX7293076 (https://www.ncbi.nlm.nih.gov/sra/SRX7293076[accn]) for the VG duplicates, and SRX7293075 (https://www.ncbi.nlm.nih.gov/sra/SRX7293075[accn]) for the duplicates of ovaries at emergence stage (included in the same file). An additional mRNA sequence was deposited independently on GenBank database under the accession number PP558207 (https://www.ncbi.nlm.nih.gov/nuccore/PP558207.1/) and it corresponded to the nucleic acid sequence coding for the vpcc31 venom protein that was not previously annotated. The transcriptomic sequencing data of the samples in this study are also available in the BIPAA repository [https://bipaa.genouest.org/is/parwaspdb] which possesses a user-friendly interface. All other data generated or analyzed during this study are included in this published article and its supplementary information files.
