# Supplementary figures and images for: Multi-omic approach to characterize the venom of the parasitic wasp Cotesia congregata (Hymenoptera: Braconidae)
Source: BMC Genomics. 2025 Apr 30;26:431. doi: 10.1186/s12864-025-11604-y (PMC12044726; doi:10.1186/s12864-025-11604-y)

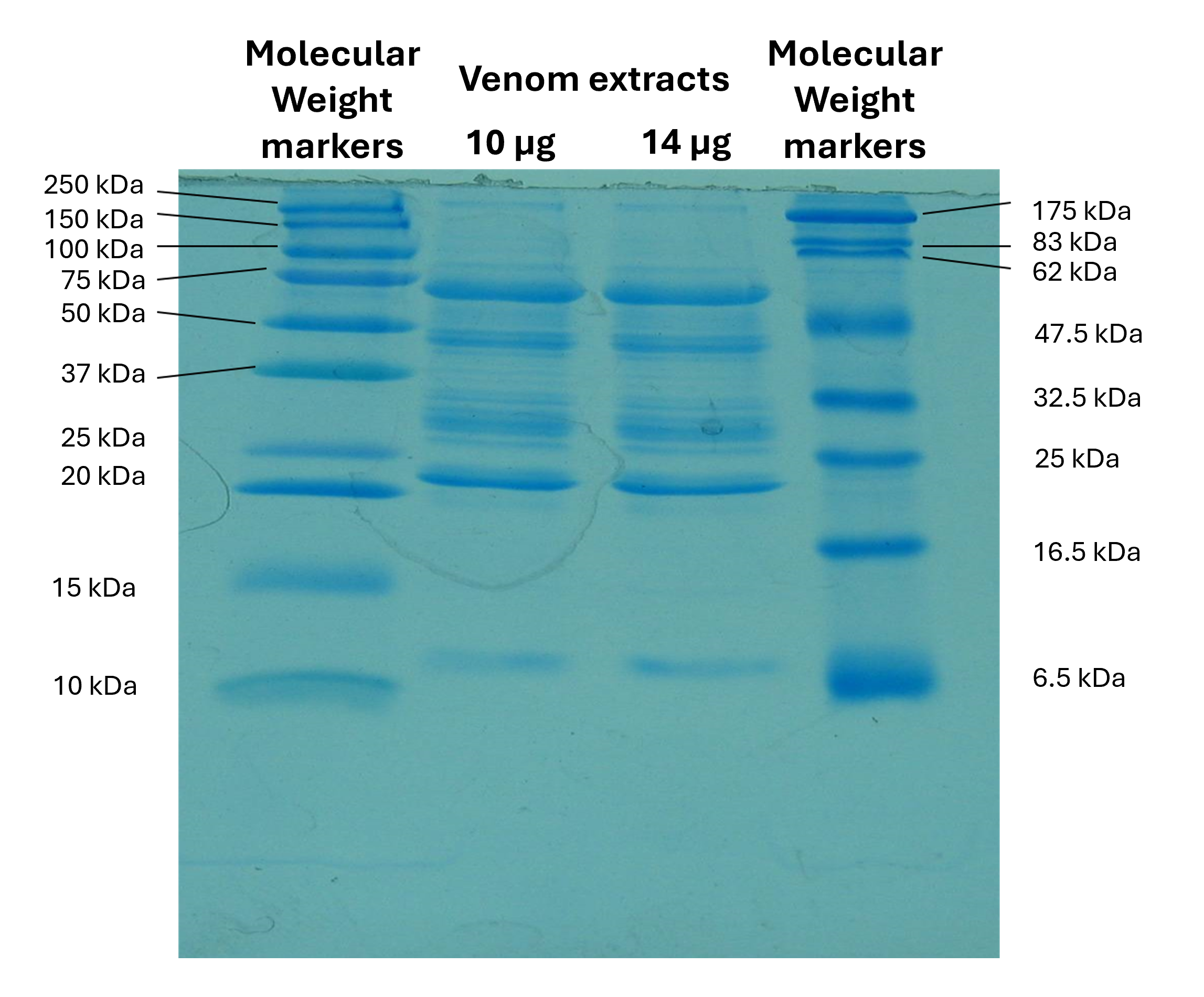

Supplement: Supplementary file 1 — Supplementary Material 1: Additional file 1: Original and unprocessed version of a 15% SDS-PAGE loaded with venom extracts of C. congregata at two different concentrations. Molecular weight markers are visible on the first and fourth line of the gel. Two samples of venom extracts, containing 10–14μg of proteins, are visible on the second and third lines of the gel [file 12864_2025_11604_MOESM1_ESM.png]

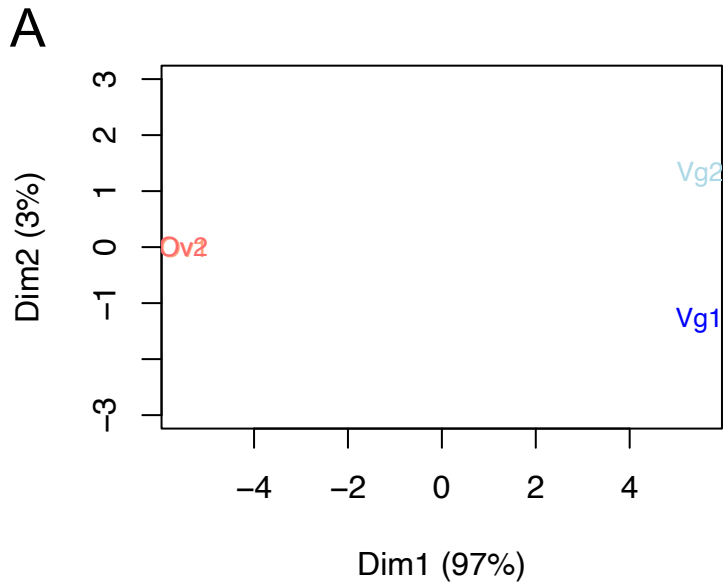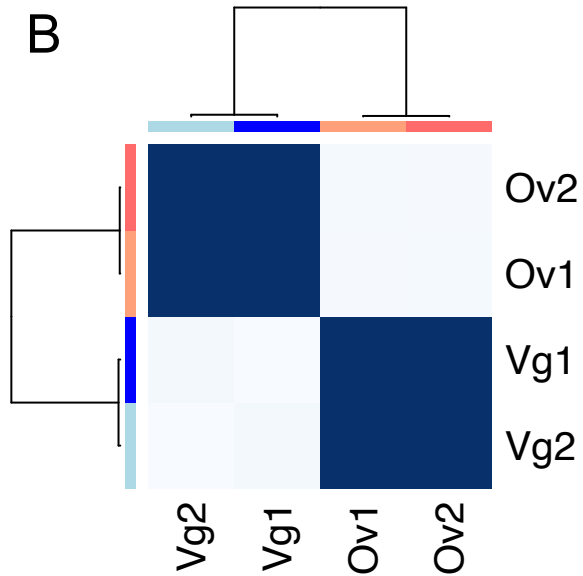

Supplement: Supplementary file 6 — Supplementary Material 6: Additional file 6: Clustering of RNA samples from ovaries and venom glands. A: Multidimensional Scaling (MDS) plot showing the relationships between ovary (Ov) and venom gland (Vg) samples based on normalized expression data; B: Correlation heatmap illustrating pairwise sample correlations based on normalized expression data. The intensity of blue shading represents the degree of correlation, with darker shades indicating higher correlation [file 12864_2025_11604_MOESM6_ESM.pdf]
