# Supplementary material for: Multi-omic approach to characterize the venom of the parasitic wasp Cotesia congregata (Hymenoptera: Braconidae)
Source: BMC Genomics. 2025 Apr 30;26:431. doi: 10.1186/s12864-025-11604-y (PMC12044726; doi:10.1186/s12864-025-11604-y)
Supplement: Supplementary file 2 — Supplementary Material 2: Additional file 2: Results of proteomic analysis. For each protein identified in the venom of C. congregata, the following information is given: total spectrum count, normalized spectral abundance factor (NSAF), total unique peptide count, total unique spectrum count and protein identification probability [file 12864_2025_11604_MOESM2_ESM.docx]

Results of proteomic analysis

| Name | Total Spectrum Count | NSAF | Total Unique Peptide Count | Total Unique Spectrum Count | Protein Identification Probability |
| --- | --- | --- | --- | --- | --- |
| 10-6 | 45 | 0,00038569 | 11 | 17 | 100 % |
| 100-6 | 112 | 0,023047 | 36 | 43 | 100 % |
| 80-4 | 79 | 0,11511 | 10 | 17 | 100 % |
| 80-6 | 135 | 0,046924 | 25 | 41 | 100 % |
| 80-10 | 71 | 0,053065 | 14 | 21 | 100 % |
| 90-3 | 127 | 0,086822 | 17 | 31 | 100 % |
| 90-9 | 78 | 0,03763 | 19 | 27 | 100 % |
| vpcc1 | 637 | 0,15734 | 56 | 97 | 100 % |
| vpcc4 | 6 | 0,0027389 | 3 | 3 | 100 % |
| vpcc5 | 22 | 0,021588 | 5 | 5 | 100 % |
| vpcc7 | 4 | 0,0049316 | 2 | 2 | 100 % |
| vpcc8 | 13 | 0,015498 | 4 | 4 | 100 % |
| vpcc12 | 41 | 0,0114 | 11 | 15 | 100 % |
| vpcc13 | 29 | 0,010652 | 11 | 13 | 100 % |
| vpcc16 | 35 | 0,018359 | 13 | 14 | 100 % |
| vpcc17 | 21 | 0,0050997 | 7 | 8 | 100 % |
| vpcc19 | 63 | 0,0072506 | 16 | 24 | 100 % |
| vpcc20 | 31 | 0,0035182 | 11 | 15 | 100 % |
| vpcc21 | 45 | 0,0084028 | 15 | 19 | 100 % |
| vpcc24 | 44 | 0,014998 | 10 | 15 | 100 % |
| vpcc29 | 59 | 0,07274 | 9 | 14 | 100 % |
| vpcc30 | 62 | 0,024982 | 19 | 22 | 100 % |
| vpcc31 | 59 | 0,047281 | 9 | 14 | 100 % |
| vpcc33 | 44 | 0,018504 | 13 | 16 | 100 % |
| vpcc34 | 89 | 0,09105 | 16 | 23 | 100 % |
| vpcc35 | 80 | 0,02885 | 21 | 28 | 100 % |
| vpcc36 | 6 | 0,0044844 | 3 | 3 | 100 % |
| vpcc37 | 15 | 0,0059607 | 7 | 7 | 100 % |
| vpcc38 | 23 | 0,0069994 | 11 | 12 | 100 % |
| vpcc39 | 8 | 0,0021135 | 4 | 4 | 100 % |

NSAF : Normalized Spectral Abundance Factor [175]
