# Supplementary material for: Multi-omic approach to characterize the venom of the parasitic wasp Cotesia congregata (Hymenoptera: Braconidae)
Source: BMC Genomics. 2025 Apr 30;26:431. doi: 10.1186/s12864-025-11604-y (PMC12044726; doi:10.1186/s12864-025-11604-y)
Supplement: Supplementary file 3 — Supplementary Material 3: Additional file 3: Description and analysis of venom proteins of unknown function and venom proteins devoid of SP. A set of 13 protein sequences deduced from genes overexpressed by VGs compared to ovaries is introduced and discussed. Secretion of these proteins by VGs was confirmed by proteomic analysis. Seven of these venom proteins possessed a predicted SP but their functions were unknown. Six of these proteins did not exhibit a predicted SP while a functional domain was found for three of them [file 12864_2025_11604_MOESM3_ESM.docx]

## Venom proteins of unknown functions

In parasitoids as in many other biological models, no conserved domain does not mean no function, as recently shown for endogenous ichnoviral genes whose functions in virus-derived particles production have been recently characterized by RNA interference assays [1]. We describe hereafter proteins possessing a predicted SP and whose presence in the venom has been confirmed by proteomic analysis but whose function remained unknown. Among these proteins, some possessed predicted domains or conserved motifs.

### Protein with a predicted collagenase-like metallopeptidase catalytic domain

The 90-9 venom protein (299 amino acids) contained a predicted collagenase-like metallopeptidase catalytic domain (InterPro: IPR024079). However, this InterPro entry designed a superfamily of conserved domains belonging to very different enzymes and proteins. Assigning a known function to 90-9 was thus difficult. A third of its tertiary structure was evocative of the catalytic domain of a peptidase M12B (ADAM/reprolysin) metalloprotease, according to the Phyre2 portal. It showed 23% of identity with the adamts-4 template (Phyre2: c2rjpC), with a 95.3% confidence index. The 90-9 protein shared 28.48 to 84.62 % of sequence identity with hypothetical proteins issued from the annotation of the genomes of *C. glomerata* (NCBI Reference Sequence: XP_044587717.1), *C. flavipes* (GenBank: UEP64265.1), *C. typhae* (GenBank: KAG8041570.1) and *M. demolitor* (NCBI Reference Sequence: XP_008546233.1). The 90-9 protein also shared 68.23 % of sequence identity with an uncharacterized protein expressed in ovaries of *C. chilonis* (GenBank: QBB01472.1). Therefore, the 90-9 protein seemed to belong to a new family of proteins of unknown function, partially sharing structural features with some metalloproteases. These proteins are encoded by genes that are specific to the Microgastrinae subfamily and can be produced by venom glands and/or ovaries.

### Proteins with tandem repeat DM9 domains

The vpcc20 (410 amino acids) and vpcc21 (412 amino acids) proteins shared 76.5 % of sequence identity. They both possessed a modified tandem repeat DM9 domain (InterPro: IPR006616) located at their C-terminal part. First identified in *Drosophila melanogaster* [2], DM9 domain containing proteins (DM9CPs) have been found in a wide range of organisms. In the pacific oyster *Crassostrea gigas*, several DM9CPs with tandem repeat are involved in innate immunity and function as mannose-specific binding pattern-recognition receptors [3, 4]. Four out of the thirteen conserved residues of the domain involved in mannose binding were observed at positions 315, 337, 348 and 388 of the mature vpcc20 protein. Interestingly, the venom of the teleost fish *Thalassophryne nattereri* contains a number of toxins, named natterins, which are DM9CPs with cytotoxic activity [5-7]. On the other hand, the C-terminal end of the first DM9 repeat of vpcc20 (PEYLQEELRLKQFEMLRGS sequence between positions 300 and 318 of the mature protein) also corresponded to a WH2 actin-binding motif (ELM accession number: ELME000313), suggesting the ability of vpcc20 to bind to different ligands. Proteins of unknown function similar to vpcc20 were found in *C. glomerata* (GenBank: KAH0551867.1), *C. typhae* (GenBank: KAG8035067.1) and *C. chilonis* (GenBank: QBB02004.1) (respectively 54.05 %, 45.38 % and 38.32 % of sequence identity with vpcc20).

### Proteins of the DUF4803 family

The Vpcc30 protein (358 amino acids) belonged to a new family of unknown functions, DUF4803 (Panther: PTHR47890, Pfam: 16061), characterized by a conserved RRY or KRY motif. Members of this family were discovered in some species of Hexapoda and Crustacea and in the venoms of *C. inanitus* (Ci-48a, Vem17 and Ci-80b proteins) [8], *C. chilonis* (Cc-Ven23) [9], *C. vestalis* (deduced protein from Unigene30513_All) [10], and *M. pulchricornis* (10 proteins whose corresponding CDS were weakly expressed by venom glands) [11]. Four proteins of this family were also detected in *M. pulchricornis* VLPs (MpVLPs) [11]. Vpcc30 exhibited 80.28 % of sequence identity with protein XP_044587790.1 from *C. glomerata* and 78.21% of sequence identity with a putative mitochondrial amidoxime-reducing component 1-like protein identified in the ovaries of *C. chilonis* (GenBank: QBB01830.1). The CDS of two other proteins related to vpcc30 and members of the PTHR47890 family in the Panther Classification system were found overexpressed by the venom glands of *C. congregata*: vpcc31 and vpcc33. Devoid of SPs, these proteins are discussed below.

### Proteins with no predicted functional domain

The 80-4 protein was 99 amino acids long. It shared 60.61 to 75.76 % of sequence identity with a group of sequences of hypothetical and uncharacterized proteins of similar lengths from *C. glomerata* (GenBank accession numbers KAH0549089.1, BAN81734.1, BAN81735.1, and KAH0549084.1 and NCBI Reference Sequence XP_044591521.1), *C. chilonis* (GenBank: QBB01859.1 and QBB01483.1) and *C. flavipes* (GenBank: UEP64256.1). The 80-4 protein was also somewhat similar to the uncharacterized protein LOC106693114 (NCBI Reference Sequence: XP_008549968.3, 31.11 % of sequence identity), found in a transcriptome of adult males from *M. demolitor* (whole body).

The vpcc16 protein (275 amino acids) shared 78.91 % of sequence identity with the hypothetical protein G9C98_007458 from *C. typhae* (GenBank: KAG8034382.1). Like 80-4 protein, no functional domain could be predicted for vpcc16.

### Cystein-rich protein

Vpcc37 was a 363 amino acids long protein containing a SP of 21 residues. The sequence contained an epidermal growth factor (EGF)-like domain (PROSITE : PS01186) characterized by six cysteine residues divided into two groups, or triades, at positions 80-93 and 208-223 of the mature protein. The vpcc37 protein shared 31.18% of sequence identity with a fibrillin-1-like protein from *C. glomerata* (NCBI Reference Sequence: XP_044579413.1) which possessed seven triades of cysteine residues forming at least 3 EGF-like domains.

Interestingly, vpcc37 contained an overall of 44 cysteine residues, representing more than 12% of all the amino acids of its sequence. The InterPro prediction program suggested the possible presence, at the N-terminal end of the mature protein, of a domain structurally related to the isoform A of the expression product of the ecdysone-inducible gene E1 (Panther classification system: PTHR39069). Proteins related to this family exhibit 24 cysteine residues at conserved positions and are produced by various arthropod species. On another hand, cysteine-rich secretory proteins (CRISPs) were found in snake venoms and venoms of cone snails and Vespidae [7]. Their presence in the venoms of distant species would apparently result from convergent evolutionary events that have selected molecules able to block ionic channels and/or to act as proteinase inhibitors. These properties rely on their particular secondary structures, maintained by several disulfide bridges assembled into cysteine knots. Cystein-rich proteins are also known factors of virulence encoded by the integrated genomes of symbiotic polydnavirus of some parasitoid wasps For example, the *Microplitis demolitor* bracovirus (MdBV) carried by the parasitoid wasp *M. demolitor* produces a protein, Egf1.0, which inhibits the phenoloxidase (PO) cascade. Egf1.0 belongs to a MdBV gene family that produces proteins with repeated identical eight-cysteine-rich domain and C-terminal repeat domains [12].

## Venom proteins devoid of SP

In the following section, we describe venom proteins whose presence in the venom has been confirmed by proteomic analysis but which are apparently devoid of predicted SP.

### Proteins with metalloprotease domains

The vpcc38 protein (474 amino acids) possessed two CUB (for complement C1r/C1s, Uegf, Bmp1) domains (InterPro: IPR000859), at positions 241-357 and 358-474 and an astacin-like metallopeptidase domain (InterPro: IPR034035), extending from positions 49 to 236. The vpcc38 protein thus belonged to zinc-dependent metalloproteases and more precisely to the astacin-like subfamily (peptidase subfamily M12A). Two peptidases from the astacin-like subfamily, Cc-Ven3 and Cc-Ven4, were previously described from the venom of the parasitoid *C. chilonis* [9].

The vpcc39 protein (546 amino acids) corresponded to another zinc-dependent metalloprotease, belonging to the M1 peptidase family. Members of this clan are membrane alanine aminopeptidases (aminopeptidases N, also designed as APN) that cleave single amino acids from the amino terminus of small peptides [13]. The vpcc39 protein’s sequence possessed a Peptidase family M1 domain (PFAM : PF01433) encompassing residues 14 to 113 and an ERAP1-like C-terminal domain (PFAM : 11838) located between positions 205 and 495. APNs were reported from venoms of snakes and predatory ants and are supposed to cause tissue damage or inflammation in envenomated animals [14, 15]. Teng and collaborators [9] identified an APN in the venom and the ovaries of *C. chilonis* which they named Cc-Ven6 (GenBank: QBB01338.1). Vpcc39 and Cc-Ven6 shared 86.68 % of sequence identity.

### Protein with no predicted functional domain

The vpcc34 protein (141 amino acids) was devoid of any known functional domain. It shared 44.09, 45.86 and 58.02 % of sequence identity with hypothetical proteins from *C. flavipes* (GenBank: UEP64252.1), *C. typhae* (GenBank: KAG8040250.1) and *C. glomerata* (NCBI Reference Sequence: XP_044581775.1).

### Protein with serpin domains

The vpcc35 protein (400 amino acids) belonged to the serpin family (InterPro: IPR000215) which includes known regulators of proteases involved in insect innate immunity [16]. Vpcc35 contained two serpin domains (InterPro: IPR042178 and IPR042185) and a conserved site (Prosite: PS00284) at positions 370 to 380, found in serpin proteins. Serpins were already reported from several parasitoid venoms, for example in *L. boulardi* [17], *Microplitis mediator* [18] and in *P. puparum* [19]. In the latter species, multiple isoforms of the PpSerpin-1 protein are expressed in the venom gland through alternative splicing [19] and some of the alternative proteins are devoid of SP. Interestingly, reports implicating serpins in zinc metalloprotease inhibition are very rare [20]. Thus, inhibition of vpcc38 and vpcc39 by vpcc35 are rather unlikely.

### Proteins of the DUF4803 family

The vpcc31 protein (389 amino acids) shared 41.37 % of sequence identity with vpcc30. The vpcc33 (343 amino acids) shared 38.49% of identity with vpcc30 and possessed the DUF4803 domain and the KRY motif found in vpcc30. It exhibited 67.27 % of sequence identity with a venom protein from *C. chilonis* (GenBank: APD15632.1), which in turn was similar to Ci-48a from the venom of *C. inanitus*.

## References

[1] Lorenzi A, Ravallec M, Eychenne M, Jouan V, Robin S, Darboux I, Legeai F, Gosselin-Grenet AS, Sicard M, Stoltz D, Volkoff A-N. RNA interference identifies domesticated viral genes involved in assembly and trafficking of virus-derived particles in ichneumonid wasps. PLoS Pathog. 2019;15:e1008210. <https://doi.org/10.1371/journal.ppat.1008210>.

[2] Ponting CP, Mott R, Bork P, Copley RR. Novel protein domains and repeats in *Drosophila melanogaster*: insights into structure, function, and evolution. Genome Res. 2001;11:1996–2008. <https://doi.org/10.1101/gr.198701>.

[3] Jiang S, Wang L, Huang M, Jia Z, Weinert T, Warkentin E, Liu C, Song X, Zhang H, Witt J, Qiu L, Peng G, Song L. DM9 Domain containing protein functions as a pattern recognition receptor with broad microbial recognition spectrum. Front Immunol. 2017;8:1607. <https://doi.org/10.3389/fimmu.2017.01607>.

[4] Jia Z, Jiang S, Wang M, Wang X, Liu Y, Lv Z, Song X, Li Y, Wang L, Song L. Identification of a novel pattern recognition receptor DM9 domain containing protein 4 as a marker for pro-hemocyte of pacific oyster *Crassostrea gigas*. Front Immunol. 2021;11:603270. <https://doi.org/10.3389/fimmu.2020.603270>.

[5] Magalhães GS, Lopes-Ferreira M, Junqueira-de-Azevedo IL, Spencer PJ, Araújo MS, Portaro FC, Ma L, Valente RH, Juliano L, Fox JW, Ho PL, Moura-da-Silva AM. Natterins, a new class of proteins with kininogenase activity characterized from *Thalassophryne nattereri* fish venom. Biochimie. 2005;87:687–99. <https://doi.org/10.1016/j.biochi.2005.03.016>.

[6] Lopes-Ferreira M, Grund LZ, Lima C. *Thalassophryne nattereri* fish venom : from the envenoming to the understanding of the immune system. J Venom Anim Toxins Incl Trop Dis. 2014;20:35. <https://doi.org/10.1186/1678-9199-20-35>.

[7] Tadokoro T, Modahl CM, Maenaka K, Aoki-Shioi N. Cysteine-Rich Secretory Proteins (CRISPs) from venomous snakes: An overview of the functional diversity in a large and underappreciated superfamily. Toxins. 2020;12:175. <https://doi.org/10.3390%2Ftoxins12030175>.

[8] Vincent B, Kaeslin M, Roth T, Heller M, Poulain J, Cousserans F, Schaller J, Poirié, M, Lanzrein B, Drezen J-M, Moreau SJM. The venom composition of the parasitic wasp *Chelonus inanitus* resolved by combined expressed sequence tags analysis and proteomic approach. BMC Genomics. 2010;11:693. <https://doi.org/10.1186/1471-2164-11-693>.

[9] Teng ZW, Xiong SJ, Xu G, Gan SY, Chen X, Stanley D, Yan ZC, Ye GY, Fang Q. Protein discovery: combined transcriptomic and proteomic analyses of venom from the endoparasitoid *Cotesia chilonis* (Hymenoptera: Braconidae). Toxins. 2017;9:135. <https://doi.org/10.3390/toxins9040135>.

[10] Zhao W, Shi M, Ye Xq; Li F, Wang X-W, Chen X-X. Comparative transcriptome analysis of venom glands from *Cotesia vestalis* and *Diadromus collaris*, two endoparasitoids of the host *Plutella xylostella*. Sci Rep. 2017;7:1298. <https://doi.org/10.1038/s41598-017-01383-2>.

[11] Gatti J-L, Belghazi M, Legeai F, Ravallec M, Frayssinet M, Robin S, Aboubakar-Souna D, Srinivasan R, Tamò, M, Poirié, M, Volkoff A-N. Proteo-trancriptomic analyses reveal a large expansion of metalloprotease-like proteins in atypical venom vesicles of the wasp *Meteorus pulchricornis* (Braconidae). Toxins. 2021;13:502. <https://doi.org/10.3390/toxins13070502>.

[12] Beck MH, Strand MR. A novel polydnavirus protein inhibits the insect prophenoloxidase activation pathway. Proc Natl Acad Sci USA. 2007;104:19267–72. <https://doi.org/10.1073/pnas.0708056104>.

[13] Rawlings ND, Barrett AJ. Evolutionary families of metallopeptidases. Methods Enzymol. 1995;248:183–228. <https://doi.org/10.1016/0076-6879(95)48015-3>.

[14] Peirera Santos P, Dias Games P, Oliveira Azevedo D, Barros E, Licursi de Oliveira L, de Oliveira Ramos HJ, Baracat-Pereira MC, Serrão JE. Proteomic analysis of the venom of the predatory ant *Pachycondyla striata* (Hymenoptera: Formicidae). Arch Insect Biochem Physiol. 2017;96:e21424. <https://doi.org/10.1002/arch.21424>.

[15] Naephrai S, Khacha-Ananda S, Pitchakarn P, Jaikang C. Composition and acute inflammatory response from *Tetraponera rufonigra* venom on RAW 264.7 macrophage cells. Toxins. 2021;13:257. <https://doi.org/10.3390/toxins13040257>.

[16] Meekins DA, Kanost MR, Michel K. Serpins in arthropod biology. Semin Cell Dev Biol. 2017;62:105–19. <https://doi.org/10.1016/j.semcdb.2016.09.001>.

[17] Colinet D, Dubuffet A, Cazes D, Moreau SJM, Drezen J-M, Poirié, M. A serpin from the parasitoid wasp *Leptopilina boulardi* targets the *Drosophila* phenoloxidase cascade. Dev Comp Immunol. 2009;33:681–9. <https://doi.org/10.1016/j.dci.2008.11.013>.

[18] Zhou L, Wang R, Lin Z, Shi S, Chen C, Jiang H, Zou Z, Lu Z. Two venom serpins from the parasitoid wasp *Microplitis mediator* inhibit the host prophenoloxidase activation and antimicrobial peptide synthesis. Insect Biochem Mol Biol. 2023;152:103895. <https://doi.org/10.1016/j.ibmb.2022.103895>.

[19] Yan Z, Fang Q, Song J, Yang L, Xiao S, Wang J, Ye G. A serpin gene from a parasitoid wasp disrupts host immunity and exhibits adaptive alternative splicing. PLoS Pathog. 2023;19:e1011649. <https://doi.org/10.1371/journal.ppat.1011649>.

[20] Ravi Ram K, Sirot LK, Wolfner MF. Predicted seminal astacin-like protease is required for processing of reproductive proteins in *Drosophila melanogaster*. Proc Natl Acad Sci USA. 2006;103:18674–9. <https://doi.org/10.1073%2Fpnas.0606228103>.
