# Supplementary material for: Multi-omic approach to characterize the venom of the parasitic wasp Cotesia congregata (Hymenoptera: Braconidae)
Source: BMC Genomics. 2025 Apr 30;26:431. doi: 10.1186/s12864-025-11604-y (PMC12044726; doi:10.1186/s12864-025-11604-y)
Supplement: Supplementary file 4 — Supplementary Material 4: Additional file 4: Description and analysis of putative venom proteins and peptides and products of genes overexpressed by venom glands. A selection of 18 additional protein sequences deduced from genes overexpressed by VGs compared to ovaries is introduced and discussed. Secretion of these proteins by VGs could not be confirmed by proteomic analysis. All putative venom proteins and peptides possessed a predicted SP, except 80-5b [file 12864_2025_11604_MOESM4_ESM.docx]

## Putative venom proteins and peptides

We introduce and discuss hereafter proteins and peptides overexpressed by venom glands and exhibiting SPs (at the exception of 80-5b) but whose presence in the venom has not been confirmed by proteomic analysis.

### Calreticulin

The venom of *C. congregata* contained vpcc2, a 404 residues long calreticulin-like protein. The mature protein displayed three calreticulin family signatures at positions 79 to 94 (Calreticulin family signature 1, PROSITE entry : PS00803), 111 to 119 (Calreticulin family signature 2, PROSITE : PS00804) and 223 to 235 (Calreticulin family repeated motif signature, PROSITE : PS00805). Vpcc2 is highly similar to calreticulins identified in the venoms of *C. vestalis* (GenBank: ANQ80539.1) and *C. rubecula* (GenBank: AAN73309.1) (98.50 and 98.00 % of sequence identity, respectively). Both of these proteins were shown to alter the immune responses of the hosts of *C. vestalis* [1] and *C. rubecula* [2]. A calreticulin-like protein from the venom of *P. hypochondriaca* appears to be responsible for modifying cellular calcium homeostasis and for inducing swelling and lysis of susceptible cells [3]. Calreticulins were also reported from the venoms or venom gland transcriptomes of several various other parasitoids: *Microctonus hyperodae* and *Microctonus aethiopoides* [4], *N. vitripennis* [5]; *Psyttalia lounsburyi* and *Psyttalia concolor* [6], *C. vestalis* [7], *Tetrastichus brontispae* [8] and *Pachycrepoideus vindemmiae* [9, 10]. In *P. puparum* and *Cotesia chilonis*, the expression of calreticulin was also observed in other tissues, like head and ovaries, in addition to its expression by venom glands [11, 12]. Calreticulins of mammals have been demonstrated to have a lectin-like activity and to play an important role in glycoprotein maturation and quality control of protein production in the endoplasmic reticulum [13].

### Proteins with serpin domains

Two closely-related putative venom proteins, vpcc23 (106 residues) and vpcc11 (122 residues) shared 50.30% of sequence identity and were not related to any known protein. The vpcc23 contained a serine protease inhibitor-like domain (InterPro: IPR036084) that usually contains ten cysteine residues forming five disulphide bonds. Only 8 cystein residues were present in the sequence of the mature vpcc23 protein. The mature sequence of vpcc11 also contained 8 cystein residues and no functional domain could be deduced from its sequence by the InterPro algorithm.

### Cystatin-like protein

The vpcc22 protein (135 amino acids) contained a cystatin-like domain (InterPro: IPR046350) and shared 75.81 to 76.30 % of sequence identity with hypothetical proteins from *C. flavipes* (GenBank: UEP64325.1), *C. typhae* (GenBank: KAG8040967.1) and *C. glomerata* (GenBank: KAH0560431.1). In *C. flavipes*, the similar protein with cystatin-like domain was expressed by teratocyte cells, in addition to a cystatin and a cystatin-like protein [14]. Cystatins are cysteine protease inhibitors that play key regulatory roles in protein degradation processes [15]. Three viral cystatins (CCBVcyst1, CCBVcyst2 and CCBVcyst3) encoded by the genome of the symbiotic polydnavirus of *C. congregata*, CCBV, are also known [16]. Their targets would be parasite-responsive cystein proteases identified in the host *Manduca sexta* [17]. It has been demonstrated, in particular wasp lineages, that some polydnavirus genes result from duplication and recruitement of cellular genes originating from the genome of their wasp host. This is the case for recent acquisitions of genes encoding sugar transporters, found in the genomes of *G. indiensis* and *G. flavicoxis* bracoviruses [18]. Vpcc22 only shared 12.3 to 13.7% of sequence identity with the CcBV cystatins. This suggests either different origins for the viral and venomous cystatins of *C. congregata*, or a common but ancient origin of these genes followed by strong sequence divergence resulting from the different selective pressures that applied to these virulence factors.

### Protein with an IAP-binding motif

The vpcc10 protein (170 amino acids) belonged to a new group of proteins of unknown function that seem specific to *Cotesia* wasps. Genes similar to its coding sequence were only found in the genomes of parasitoids of the genus *Cotesia* (including *C. glomerata*, *C. typhae*, *C.flavipes*, *C. rubecula*, *C. sesamiae kitale* and *C. vestalis*). For instance, the *C. glomerata* genome coded for three similar hypothetical proteins sharing 84.30 to 89.17 % of sequence identity with vpcc10 (NCBI Reference Sequence: XP_044576782.1, XP_044576793.1 and KAH0567802.1). A similar hypothetical protein (GenBank: KAG8037267.1) that shared 76.47 % of sequence identity with vpcc10 was encoded by *C. flavipes* genome. Interestingly, the sequence of vpcc10 contained an Inhibitor of Apoptosis Proteins (IAP)-binding motif (ELM accession number: ELME000285) that specifically binds to baculoviral IAP repeat domain. This IAP-binding motif is usually located at the N-terminal regions of caspases and IAP-antagonizing proteins, which suggests that vpcc10 could have a pro-apoptotic function. However, the motif was observed between positions 1 to 5 of the precursor molecule and hence at the N-terminal end of its predicted SP. This would hamper its chances to have a regulatory function if the protein was secreted, due to the expected cleavage of the N-terminal part of the precursor protein. The vpcc10 also contained several tyrosine-containing motifs, susceptible to form phosphotyrosine ligands bound by Src Homology 2 (SH2) domains, the main binding domain for tyrosine kinase phosphorylation signaling events. Such motifs can be found at positions 39 to 42 (LIG_SH2_GRB2like motif, ELM accession number: ELME000084), 60 to 64 (LIG_SH2_NCK_1 motif, ELME000474), 64 to 68, 69 to 73 and 73 to 77 (LIG_SH2_CRK motifs, ELME000458) of the precursor vpcc10 protein. According to the ELM server, the probability to find sequences of amino acids corresponding by chance to these motifs was low (respectively 3.175e-04, 8.293e-04 and 1.515e-03). Thus, vpcc10 may have the ability to bind to SH2 domain containing proteins and to modify their function and/or activity.

### Copper/Zinc superoxide dismutase

The venom glands of *C. congregata* overexpressed a gene encoding a Copper/Zinc superoxide dismutase (SOD) (InterPro: IPR024134). This family of antioxidative stress enzymes are well known in arthropod venoms. They have notably been described from the venoms of the Indian scorpion *Heterometrus fulvipes* [19], the Egyptian honeybee *Apis mellifera lamarkii* [20] and in the parasitoid species *L. boulardi, L. heterotoma* and *Tetrastichus brontispae* [21, 22]. The venom glands of *C. vestalis* also overexpress a SOD [7]. Venom SODs would directly act by interfering with the melanization pathway of envenomated arthropods, or indirectly by protecting venom components from oxidative damages during their storage in the venom reservoir. Vpcc40, the SOD produced by the venom glands of *C. congregata*, possessed a N-terminal SP and two signature patterns characteristic of this family of enzymes (PROSITE entry: PS00087). The first signature was located from positions 43 to 53 of the mature protein and contained two histidine residues that bind the copper or zinc atom. The second signature was located N-terminally, between positions 137 and 148, and contained a cysteine residue involved in a disulfide bond with another cysteine. The enzyme seemed to possess all required active sites to be fully active in the venom or to protect the venom gland.

### Protein disulphide isomerase-like protein

Vpcc28 belonged to the protein disulphide isomerase (PDI) family (InterPro: IPR005792). It contained three thioredoxin-like active sites. Interestingly, PDI family members are known to function as chaperones and redox-catalysts in eukaryotes [23]. One of these members, ERp57, has been shown to bind to calreticulin and to intervene in the proper folding of glycoproteins. It is plausible that vpcc28 interacts with calreticulin and/or vpcc2 in the lumen of the endoplasmic reticulum and is involved in the formation and reshuffling of the disulfide bridges of venom proteins. In the parasitoid wasp *Asobara japonica*, a thioredoxin peroxidase produced by the ovaries is supposed to antagonize the toxic effects of the venom of this species [24] that would otherwise kill the envomated host.

### Putative venom peptides

Since we have concentrated our efforts on identifying venomous proteins of *C. congregata*, we excluded venomous peptides from the analysis, due to size exclusion during the SDS-PAGE migration of venom extracts. However, a number of genes encoding peptides were found expressed at high levels in venom glands, and most of the peptides deduced from these genetic sequences possessed a predicted SP. Their presence in the venom of *C. congregata* is thus probable but remains hypothetical. They are good candidates to consider for reconstructing the peptidomic fraction of *C. congregata*’s venom.

The most expressed gene in venom glands encoded a 64 residues polypeptide (80-5a peptide). Its amino acid sequence started with a predicted SP of 23 residues long, followed by 40 residues forming a non-cytoplasmic domain. The only similar sequences available in databases were from *C. rubecula* (Vn4.6 venom protein, UniProtKB/Swiss-Prot accession number Q8WQK0.2), *C. vestalis* (TSVP-8 polypeptide, GenBank accession number AGI44424.1), and *C. glomerata* (KQX54_002300 hypothetical protein, GenBank accession number KAH0557254.1). The overall percentage of identity between the 80-5a sequence and the other sequences reached 73.85 % with Vn4.6, 51.47 % with KQX54_002300 and 46.27 % with TSVP-8. Remarkably, most identities were found in the SPs of the precursor polypeptides: the SP of 80-5a was fully identical to the SP of Vn4.6, 91.3 % identical to the SP of KQX54_002300 and 78.26% identical to the SP of TSVP-8. The sequence of KQX54_002300 was also much longer (275 amino acids including the SP) than those of the others (64 to 71 amino acids). This suggests a relatively recent gene acquisition by an ancestor of the genus *Cotesia*, followed by diversifying selection on parts of the genes coding for the secreted peptides, while a more conservative selection probably occurred on their SPs. In *C. rubecula*, the Vn4.6 protein interferes with the activation of the host hemolymph prophenoloxidase [25]. In *C. vestalis* it has been suggested that the homolog of Vn4.6 might help the parasitoid to escape the host’s immune response [7].

A second gene, encoding the 80-5b peptide, was highly expressed in venom glands compared to ovaries of *C. congregata*. The deduced sequence contained a predicted SP of 20 amino acids, according to SignalP 4.0 followed by a sequence of 14 amino acids. However, this prediction was not confirmed by the 6.0 version of SignalP, which casted doubts on its ability to be secreted by a classical pathway. According to the InterPro server, the entire sequence of 80-5b would in fact correspond to a membrane-bound polypeptide, with its N-terminal end (the 20 amino acids “SP”) predicted to be embedded in the membrane, while its C-terminal end (the 14 amino acids sequence) was predicted to be outside the membrane, in the extracellular region of the producing cell. The secretion of the mature peptide by the venom glands is thus possible but would require a cleavage mediated by a peptidase. In an attempt to explore the functions of 80-5b, we have produced, purified and tested a synthetic peptide, corresponding to the potentially cleaved 80-5b peptide, against *Escherichia coli* and *Micrococcus luteus* colonies. It did not show any measurable antibacterial activity (data not shown).

Three other polypeptides shorter than 80 amino acids long were overexpressed by venom glands and possessed predicted SPs: Vpcc9 (46 residues), Vpcc6 (76 residues) and Vpcc14 (78 residues). They were all of unknown function. A forth peptide, Vpcc15 (36 residues), had a prediction score for the presence of a SP of only 0.521, returned by the SignalP 6.0 algorithm. The sequences of Vpcc15, Vpcc9 and Vpcc14 did not return any significant homology with known sequences after similarity searches using the BLASTP program. The sequence of Vpcc6 had only two significant matches with hypothetical proteins KQX54_020508 (GenBank: KAH0550679.1) and KQX54_020509 (GenBank: KAH0550680.1) from *C. glomerata* (68.92 % and 64.06 % of sequence identity, respectively).

## Products of genes overexpressed by venom glands

In this last part of our analysis, we have gathered interesting gene products which are overexpressed by venom glands of *C. congregata*. None of them possessed a SP and proteomic analysis did not bring any evidence of their secretion in the venom.

### Protein with multifunctional binding motifs

The vpcc27 protein (324 amino acids) had no predicted conserved domain. Its peptidic sequence shared 27.17 to 29.65 % of sequence identity with hypothetical proteins of unknown functions (NCBI Reference Sequence: XP_006811633.1 and GenBank: KAH3707592.1) encoded respectively by the genomes of two distant aquatic organisms, *Saccoglossus kowalevskii* (Enteropneusta: Harrimaniidae) and *Dreissena polymorph*a (Bivalvia: Dreissenidae). Interestingly, vpcc27 exhibited a repeated motif of eleven residues (RTLSDGTCSII) that could correspond to several eukaryotic linear motifs, according to the ELM server. Ten out of the eleven residues of this motif (RTLSDGTCSI) formed a 14-3-3 binding phosphopeptide motif (ELM accession number: ELME000417), able to bind with a low micromolar affinity to 14-3-3 regulatory proteins. The “SDGTCSI” pattern could correspond to a Casein kinase 1 (CK1) Phosphorylation site (ELM accession number: ELME000063), while the “GTCSIIRT” sequence was recognized as a Glycogen Synthase kinase 3 (GSK3) phosphorylation site ((ELM accession number: ELME000053). The “DGTCSII” part of the motif corresponded to a phosphothreonine containing peptide (ELM accession number: ELME000052) recognized by forkhead-associated (FHA) domains of regulatory proteins. The “GTCSIIR” heptapeptide was recognized as a Polo-like kinase phosphosite that, once phosphorylated, could interact with acidophilic polo-like kinases. Finally, the “TLSDG” part of the motif also corresponded to a Caspase-3 or Caspase-7 cleavage site (ELM accession number: ELME000321).

### Hyaluronidase-like protein

Venom glands of *C. congregata* also overexpressed a gene encoding vpcc41, a hyaluronidase-like protein (340 amino acids) that belonged to the Glycoside hydrolase family 56 (InterPro: IPR001329). Hyaluronidases are well studied venom components of arthropods and snakes, first described from the venom of the tarantula *Dugesiella hentzi* [26]. In addition, one of the main allergens of the venom of *A. mellifera*, Api m2, is a venom hyaluronidase [27; 28]. In Braconidae, venom hyaluronidases were reported from *C. inanitus* [29]. Hyaluronidase genes are also expressed by the venom gland filament of *M. pulchricornis* [30] but it is not clear if the corresponding gene product is secreted in the venom. These enzymes would also be present, sometimes at barely detectable levels of activity, in the venoms of hymenopteran species belonging to Ichneumonidae [31], Eupelmidae [32], Eulophidae [33], Pteromalidae [34], Ampulicidae [35], Eumenidae [36], Vespidae [37], Pompilidae [38], Mutilidae [39] and Formicidae [40, 41]. Venom hyaluronidases would act as spreading factors, facilitating the action of venom toxins [42]. In *C. congregata*, the vpcc41 gene was significantly overexpressed by venom glands compared to ovaries but the protein, which lacked a predicted SP, was not detected in venom. It seems thus that vpcc41 is produced by the venom gland but not secreted at detectable levels.

### Peptide of unknown function

Finally, the polypeptide Vpcc26 (50 residues) was also encoded by a gene overexpressed in venom glands compared to ovaries, and lacked a predicted SP. No similar sequence was found in the databases and its function is therefore unknown to date.

## References

[1] Cha WH, Kim Y, Lee D-W. Calreticulin in *Cotesia plutellae* suppresses immune response of *Plutella xylostella* (L.). J Asia Pac Entomol. 2015;18:27–31. <https://doi.org/10.1016/j.aspen.2014.11.001>.

[2] Zhang G, Schmidt O, Asgari S. A calreticulin-like protein from endoparasitoid venom fluid is involved in host hemocyte inactivation. Dev Comp Immunol. 2006;30:756–64. <https://doi.org/10.1016/j.dci.2005.11.001>.

[3] Rivers DB, Dani MP, Richards EH. The mode of action of venom from the endoparasitic wasp *Pimpla hypochondriaca* (Hymenoptera: Ichneumonidae) involves Ca+2-dependent cell death pathways. Arch Insect Biochem Physiol. 2009;71:173–90. <https://doi.org/10.1002/arch.20314>.

[4] Crawford AM, Brauning R, Smolenski G, Ferguson C, Barton D, Wheeler TT, McCulloch A. The constituents of *Microctonus* sp. parasitoid venoms. Insect Mol Biol. 2008;17:313–24. <https://doi.org/10.1111/j.1365-2583.2008.00802.x>.

[5] Siebert AL, Wheeler D, Werren JH A new approach for investigating venom function applied to venom calreticulin in a parasitoid wasp. Toxicon. 2015;107:304–16. <https://doi.org/10.1016/j.toxicon.2015.08.012>.

[6] Mathé-Hubert H, Colinet D, Deleury E, Belghazi M, Ravallec M, Poulain J, Dossat C, Poirié, M, Gatti JL. Comparative venomics of *Psyttalia lounsburyi* and *P. concolor*, two olive fruit fly parasitoids : a hypothetical role for a GH1 β-glucosidase. Sci Rep. 2016;6:35873. <https://doi.org/10.1038/srep35873>.

[7] Zhao W, Shi M, Ye Xq; Li F, Wang X-W, Chen X-X. Comparative transcriptome analysis of venom glands from *Cotesia vestalis* and *Diadromus collaris*, two endoparasitoids of the host Plutella xylostella. Sci Rep. 2017;7:1298. <https://doi.org/10.1038/s41598-017-01383-2>.

[8] Tang BZ, Meng E, Zhang HJ, Zhang XM, Asgari S, Lin YP, Lin YY, Peng ZQ, Qiao T, Zhang XF, Hou YM. Combination of label-free quantitative proteomics and transcriptomics reveals intraspecific venom variation between the two strains of *Tetrastichus brontispae*, a parasitoid of two invasive beetles. J Proteomics. 2019;192:37–53. <https://doi.org/10.1016/j.jprot.2018.08.003>.

[9] Yang L, Yang Y, Liu MM, Yan ZC, Qiu LM, Fang Q, Wang F, Werren JH, Ye GY. Identification and comparative analysis of venom proteins in a pupal ectoparasitoid, *Pachycrepoideus vindemmiae*. Front Physiol. 2020;11:9. <https://doi.org/10.3389/fphys.2020.00009>.

[10] Yang L, Wang B, Qiu L, Wan B, Yang Y, Liu M, Wang F, Fang Q, Stanley DW, Ye G. Functional characterization of a venom protein calreticulin in the ectoparasitoid *Pachycrepoideus vindemmiae*. Insects. 2020;11:29. <https://doi.org/10.3390/insects11010029>.

[11] Teng ZW, Xiong SJ, Xu G, Gan SY, Chen X, Stanley D, Yan ZC, Ye GY, Fang Q. Protein discovery: combined transcriptomic and proteomic analyses of venom from the endoparasitoid *Cotesia chilonis* (Hymenoptera: Braconidae). Toxins. 2017;9:135. <https://doi.org/10.3390/toxins9040135>.

[12] Wang L, Fang Q, Qian C, Wang F, Yu XQ, Ye G. Inhibition of host cell encapsulation through inhibiting immune gene expression by the parasitic wasp venom calreticulin. Insect Biochem Mol Biol. 2013;43:936–46. <https://doi.org/10.1016/j.ibmb.2013.07.010>.

[13] Peterson JR, Ora A, Van PN, Helenius A. Transient lectin-like association of calreticulin with folding intermediates of cellular and viral glycoproteins. Mol Biol Cell. 1995;6:1173–84. <https://doi.org/10.1091/mbc.6.9.1173>.

[14] Pinto CPG, Walker AA, Robinson SD, King GF, Rossi GD. Proteotranscriptomics reveals the secretory dynamics of teratocytes, regulators of parasitization by an endoparasitoid wasp. J Insect Physiol. 2022;139:104395. <https://doi.org/10.1016/j.jinsphys.2022.104395>.

[15] Kordis D, Turk V. Phylogenomic analysis of the cystatin superfamily in eukaryotes and prokaryotes. BMC Evolutionary Biology. 2009;9:266. <https://doi.org/10.1186/1471-2148-9-266>.

[16] Serbielle C, Chowdhury S, Pichon S, Dupas S, Lesobre J, Purisima EO, Drezen J-M, Huguet E. Viral cystatin evolution and three-dimensional structure modeling: a case of directional selection acting on a viral protein involved in a host-parasitoid interaction. BMC Biol. 2008;6:38. <https://doi.org/10.1186/1741-7007-6-38>.

[17] Serbielle C, Moreau SJM, Veillard F, Voldoire E, Bézier A, Mannucci MA, Volkoff A-N, Drezen J-M, Lalmanach G, Huguet E. Identification of parasite-responsive cysteine proteases in *Manduca sexta*. Biol Chem. 2009;390:493–502. <https://doi.org/10.1515/bc.2009.061>.

[18] Desjardins CA, Gundersen-Rindal DE, Hostetler JB, Tallon LJ, Fadrosh DW, Fuester RW, Pedroni MJ, Haas BJ, Schatz MC, Jones KM, Crabtree J, Forberger H, Nene V. Comparative genomics of mutualistic viruses of *Glyptapanteles* parasitic wasps. Genome Biol. 2008;9:R183. <https://doi.org/10.1186/gb-2008-9-12-r183>.

[19] Ramanaiah M, Venkaiah B. Characterization of superoxide dismutase from south Indian scorpion venom. Biochem Int. 1992; 26:113–23. <https://pubmed.ncbi.nlm.nih.gov/1616488/>.

[20] Abdel-Monsef MM, Darwish DA, Zidan HA, Hamed AA, Ibrahim MA. Characterization, antimicrobial and antitumor activity of superoxide dismutase extracted from Egyptian honeybee venom (*Apis mellifera lamarckii*). J Genet Eng Biotechnol. 2023;21:21. <https://doi.org/10.1186%2Fs43141-023-00470-4>.

[21] Colinet D, Cazes D, Belghazi M, Gatti JL, Poirié, M. Extracellular superoxide dismutase in insects: characterization, function, and interspecific variation in parasitoid wasp venom. J Biol Chem. 2011;286:40110–21. <https://doi.org/10.1074/jbc.m111.288845>.

[22] Liu NY, Xu ZW, Yan W, Ren XM, Zhang ZQ, Zhu JY. Venomics reveals novel ion transport peptide-likes (ITPLs) from the parasitoid wasp *Tetrastichus brontispae*. Toxicon. 2018;141:88–93. <https://doi.org/10.1016/j.toxicon.2017.11.008>.

[23] Turano C, Coppari S, Altieri F, Ferraro A. Proteins of the PDI family: unpredicted non-ER locations and functions. J Cell Physiol. 2002;193:154–163. <https://doi.org/10.1002/jcp.10172>.

[24] Mabiala-Moundoungou ADN. Mechanisms and agents of the virulence in Hymenoteran braconid parasitoids of the *Asobara* genus. PhD Thesis, University of Picardie Jules Verne, Amiens, France, 2009. <https://theses.fr/2009AMIE0109>.

[25] Asgari S, Zareie R, Zhang G, Schmidt O. Isolation and characterization of a novel venom protein from an endoparasitoid, *Cotesia rubecula* (Hym: Braconidae). Arch Insect Biochem Physiol. 2003;53:92–100. <https://doi.org/10.1002/arch.10088>.

[26] Schanbacher FL, Lee CK, Wilson IB, Howell DE, Odell GV. Purification and characterization of tarantula, *Dugesiella hentzi* (girard) venom Hyaluronidase. Comp Biochem Physiol B. 1973;44:389–96. <https://doi.org/10.1016/0305-0491(73)90012-6>.

[27] Gmachl M, Kreil G. Bee venom hyaluronidase is homologous to a membrane protein of mammalian sperm. Proc Natl Acad Sci USA. 1993; 90:3569–73. <https://doi.org/10.1073/pnas.90.8.3569>.

[28] Matysiak J, Hajduk J, Pietrzak Ł, Schmelzer CE, Kokot ZJ. Shotgun proteome analysis of honeybee venom using targeted enrichment strategies. Toxicon. 2014;90:255–64. <https://doi.org/10.1016/j.toxicon.2014.08.069>.

[29] Vincent B, Kaeslin M, Roth T, Heller M, Poulain J, Cousserans F, Schaller J, Poirié, M, Lanzrein B, Drezen J-M, Moreau SJM. The venom composition of the parasitic wasp *Chelonus inanitus* resolved by combined expressed sequence tags analysis and proteomic approach. BMC Genomics. 2010;11:693. <https://doi.org/10.1186/1471-2164-11-693>.

[30] Quicke DLJ, Butcher BA. Review of venoms of non-polydnavirus carrying ichneumonoid wasps. Biology. 2021;10:50. <https://doi.org/10.3390/biology10010050>.

[31] Dorémus T, Urbach S, Jouan V, Cousserans F, Ravallec M, Demettre E, Wajnberg E, Poulain J, Azéma-Dossat C, Darboux I, Escoubas J-M, Colinet D, Gatti JL, Poirié, M, Volkoff A-N. Venom gland extract is not required for successful parasitism in the polydnavirus-associated endoparasitoid *Hyposoter didymator* (Hym. Ichneumonidae) despite the presence of numerous novel and conserved venom proteins. Insect Biochem Mol Biol. 2013;43:292–307. <https://doi.org/10.1016/j.ibmb.2012.12.010>.

[32] Doury G, Bigot Y, Periquet G. Physiological and biochemical analysis of factors in the female venom gland and larval salivary secretions of the ectoparasitoid wasp *Eupelmus orientalis*. J Insect Physiol. 1997; 43:69–81.<https://doi.org/10.1016/S0022-1910(96)00053-4>.

[33] Nakamatsu Y, Tanaka T. Venom of *Euplectrus separatae* causes hyperlipidemia by lysis of host fat body cells. J Insect Physiol. 2004;50:267–75. <https://doi.org/10.1016/j.jinsphys.2003.12.005>.

[34] Perkin LC, Friesen KS, Flinn PW, Oppert B. Venom gland components of the ectoparasitoid wasp, *Anisopteromalus calandrae*. J Venom Res. 2015;6:19–37. <https://pubmed.ncbi.nlm.nih.gov/26998218>.

[35] Arvidson R, Kaiser M, Lee SS, Urenda JP, Dail C, Mohammed H, Nolan C, Pan S, Stajich JE, Libersat F, Adams ME. Parasitoid jewel wasp mounts multipronged neurochemical attack to hijack a host brain. Mol Cell Proteomics. 2019;18:99–114. <https://doi.org/10.1074/mcp.ra118.000908>.

[36] Baek JH, Lee SH. Isolation and molecular cloning of venom peptides from *Orancistrocerus drewseni* (Hymenoptera: Eumenidae). Toxicon. 2010;55:711–8. <https://doi.org/10.1016/j.toxicon.2009.10.023>.

[37] Lee SH, Baek JH, Yoon KA. Differential properties of venom peptides and proteins in solitary vs. social hunting wasps. Toxins. 2016;8:32. <https://doi.org/10.3390%2Ftoxins8020032>.

[38] Huicab-Uribe MA, Verdel-Aranda K, Martínez-Hernández A, Zamudio FZ, Jiménez-Vargas JM, Lara-Reyna J. Molecular composition of the paralyzing venom of three solitary wasps (Hymenoptera: Pompilidae) collected in southeast Mexico. Toxicon. 2019;168:98–102. <https://doi.org/10.1016/j.toxicon.2019.06.224>.

[39] Jensen T, Walker AA, Nguyen SH, Jin AH, Deuis JR, Vetter I, King GF, Schmidt JO, Robinson SD. Venom chemistry underlying the painful stings of velvet ants (Hymenoptera: Mutillidae). Cell Mol Life Sci. 2021;78:5163–77. <https://doi.org/10.1007/s00018-021-03847-1>.

[40] Schmidt JO, Blum MS, Overal WL. Comparative enzymology of venoms from stinging Hymenoptera. Toxicon. 1986;24:907–21. <https://doi.org/10.1016/0041-0101(86)90091-7>.

[41] Matuszek MA, Hodgson WC, King RG, Sutherland SK. Some enzymic activities of two Australian ant venoms: a jumper ant *Myrmecia pilosula* and a bulldog ant *Myrmecia pyriformis*. Toxicon. 1994; 32:1543–9. <https://doi.org/10.1016/0041-0101(94)90313-1>.

[42] Bordon KC, Wiezel GA, Amorim FG, Arantes EC. Arthropod venom Hyaluronidases: biochemical properties and potential applications in medicine and biotechnology. J Venom Anim Toxins Incl Trop Dis. 2015;21:43. <https://doi.org/10.1186%2Fs40409-015-0042-7>.
