# Supplementary material for: Multi-omic approach to characterize the venom of the parasitic wasp Cotesia congregata (Hymenoptera: Braconidae)
Source: BMC Genomics. 2025 Apr 30;26:431. doi: 10.1186/s12864-025-11604-y (PMC12044726; doi:10.1186/s12864-025-11604-y)
Supplement: Supplementary file 5 — Supplementary Material 5: Additional file 5: List of putative venom proteins and peptides and products of genes overexpressed by venom glands. Name, sequence length, theoretical molecular weight and isoelectric point, level of overexpression and SP prediction are given for each gene product [file 12864_2025_11604_MOESM5_ESM.docx]

**Additional file 4** List of putative venom proteins and peptides and products of genes overexpressed by venom glands

| **Category** | **Name** | **Genbank accession number** | **Sub-category** | **Sequence length (number of amino acids)** | **Theoretical molecular weight (Da)** | **Theoretical pI** | **Level of overexpression in venom glands compared to ovaries (Fold change)** | **Presence of a predicted signal peptide** | **SignalP 6.0 score for "Other"** | **SignalP 6.0 score for SP** | **Cleavage site position and probability** | **Type of SP** |
| --- | --- | --- | --- | --- | --- | --- | --- | --- | --- | --- | --- | --- |
| Putative venom proteins and peptides | 80-5a | CAD6241156.1 | Putative venom peptides | 64 | 7176.31 | 4.46 | 1693151,328 | yes | 0.000220 | 0.999742 | CS pos: 23-24. Pr: 0.9794 | Sec/SPI |
| Putative venom proteins and peptides | 80-5b | CAD6227393.1 | Putative venom peptides | 34 | 3818.75 | 9.40 | 380289,2539 | no | 0.950692 | 0.049306 | NA | none |
| Putative venom proteins and peptides | vpcc2 | CAD6240478.1 | Calreticulin | 404 | 46779.79 | 4.37 | 14,0912219 | yes | 0.000191 | 0.999802 | CS pos: 18-19. Pr: 0.9803 | Sec/SPI |
| Putative venom proteins and peptides | vpcc6 | CAD6237083.1 | Putative venom peptides | 76 | 8540.74 | 4.91 | 593334,4626 | yes | 0.000214 | 0.999757 | CS pos: 21-22. Pr: 0.9774 | Sec/SPI |
| Putative venom proteins and peptides | vpcc9 | CAD6242943.1 | Putative venom peptides | 46 | 5105.05 | 4.70 | 26,1697989 | yes | 0.000436 | 0.999537 | CS pos: 21-22. Pr: 0.4684 | Sec/SPI |
| Putative venom proteins and peptides | vpcc10 | CAD6230760.1 | Protein with an IAP-binding motif | 170 | 19776.11 | 4.68 | 28597,88528 | yes | 0.000434 | 0.999544 | CS pos: 17-18. Pr: 0.9557 | Sec/SPI |
| Putative venom proteins and peptides | vpcc11 | CAD6204021.1 | Proteins with serpin domains | 122 | 14130.84 | 4.93 | 5063,47647 | yes | 0.000247 | 0.999732 | CS pos: 19-20. Pr: 0.9784 | Sec/SPI |
| Putative venom proteins and peptides | vpcc14 | CAD6230301.1 | Putative venom peptides | 78 | 8827.13 | 6.11 | 72247,23043 | yes | 0.000187 | 0.999784 | CS pos: 20-21. Pr: 0.9814 | Sec/SPI |
| Putative venom proteins and peptides | vpcc15 | CAD6222021.1 | Putative venom peptides | 36 | 3917.80 | 8.25 | 32813,99557 | yes | 0.478958 | 0.521039 | CS pos: 20-21. Pr: 0.4322 | Sec/SPI |
| Putative venom proteins and peptides | vpcc18 | CAD6231746.1 | Histidine phosphatase superfamily | 370 | 42905.59 | 8.85 | 20882,78193 | yes | 0.002559 | 0.997433 | CS pos: 21-22. Pr: 0.9632 | Sec/SPI |
| Putative venom proteins and peptides | vpcc22 | CAD6233947.1 | Cystatin-like protein | 135 | 15461.96 | 5.12 | 3823,992276 | yes | 0.000202 | 0.999779 | CS pos: 25-26. Pr: 0.9793 | Sec/SPI |
| Putative venom proteins and peptides | vpcc23 | CAD6240000.1 | Proteins with serpin domains | 106 | 12817.38 | 4.86 | 4094,935768 | yes | 0.000187 | 0.999781 | CS pos: 20-21. Pr: 0.9791 | Sec/SPI |
| Putative venom proteins and peptides | vpcc28 | CAD6221876.1 | Protein disulphide isomerase-like protein | 500 | 56997.07 | 4.94 | 1159,150878 | yes | 0.000275 | 0.999718 | CS pos: 19-20. Pr: 0.9749 | Sec/SPI |
| Putative venom proteins and peptides | vpcc40 | CAD6215548.1 | Copper/Zinc superoxide dismutase | 206 | 21325.91 | 6.33 | 359,4065164 | yes | 0.000466 | 0.999487 | CS pos: 17-18. Pr: 0.9390 | Sec/SPI |
| Products of genes overexpressed by venom glands | vpcc25 | CAD6204020.1 | Phosphodiesterases-like proteins of non eukaryotic origins | 346 | 39589.13 | 6.39 | 1477,169644 | yes | 0.406003 | 0.593981 | CS pos: 24-25. Pr: 0.4343 | Sec/SPI |
| Products of genes overexpressed by venom glands | vpcc26 | CAD6222127.1 | Peptide of unknown function | 50 | 5663.78 | 9.49 | 438,2662028 | no | 0.979221 | 0.020790 | NA | none |
| Products of genes overexpressed by venom glands | vpcc27 | CAD6235636.1 | Protein with multifunctional binding motifs | 324 | 34743.50 | 7.14 | 1632,638371 | no | 0.667796 | 0.332203 | NA | none |
| Products of genes overexpressed by venom glands | vpcc41 | CCQ71107.1 | Hyaluronidase-like protein | 340 | 39498.15 | 9.35 | 132,2044243 | no | 1.000075 | 0.000000 | NA | none |
